# Independent effects of dietary fat and sucrose content on chondrocyte metabolism and osteoarthritis pathology in mice

**DOI:** 10.1242/dmm.034827

**Published:** 2018-08-31

**Authors:** Elise L. Donovan, Erika Barboza Prado Lopes, Albert Batushansky, Mike Kinter, Timothy M. Griffin

**Affiliations:** 1 Aging and Metabolism Research Program, Oklahoma Medical Research Foundation (OMRF), Oklahoma City, OK 73104, USA; 2 Department of Geriatric Medicine, Reynolds Oklahoma Center on Aging, University of Oklahoma Health Sciences Center, Oklahoma City, OK 73104, USA; 3 Department of Biochemistry and Molecular Biology and Department of Physiology, University of Oklahoma Health Sciences Center, Oklahoma City, OK 73104, USA

**Keywords:** Osteoarthritis, Obesity, Cartilage, Metabolism, High-fat diet, Mouse

## Abstract

Obesity is one of the most significant risk factors for knee osteoarthritis. However, therapeutic strategies to prevent or treat obesity-associated osteoarthritis are limited because of uncertainty about the etiology of disease, particularly with regard to metabolic factors. High-fat-diet-induced obese mice have become a widely used model for testing hypotheses about how obesity increases the risk of osteoarthritis, but progress has been limited by variation in disease severity, with some reports concluding that dietary treatment alone is insufficient to induce osteoarthritis in mice. We hypothesized that increased sucrose content of typical low-fat control diets contributes to osteoarthritis pathology and thus alters outcomes when evaluating the effects of a high-fat diet. We tested this hypothesis in male C57BL/6J mice by comparing the effects of purified diets that independently varied sucrose or fat content from 6 to 26 weeks of age. Outcomes included osteoarthritis pathology, serum metabolites, and cartilage gene and protein changes associated with cellular metabolism and stress-response pathways. We found that the relative content of sucrose versus cornstarch in low-fat iso-caloric purified diets caused substantial differences in serum metabolites, joint pathology, and cartilage metabolic and stress-response pathways, despite no differences in body mass or body fat. We also found that higher dietary fat increased fatty acid metabolic enzymes in cartilage. The findings indicate that the choice of control diets should be carefully considered in mouse osteoarthritis studies. Our study also indicates that altered cartilage metabolism might be a contributing factor to how diet and obesity increase the risk of osteoarthritis.

## Abstract (precis)

**Summary:** The contribution of metabolic factors to obesity-associated knee osteoarthritis is uncertain. Here, we show how dietary fat and sucrose independently alter cartilage metabolic enzymes and osteoarthritis pathophysiology in mice.

## INTRODUCTION

Obesity is a major risk factor for knee osteoarthritis (OA) (Deshpande et al., 2016; Felson et al., 1988), doubling the lifetime risk compared with individuals with a body mass index (BMI) below 25 (Murphy et al., 2008). Biomechanical, inflammatory and metabolic factors are all believed to contribute to obesity-associated OA pathology (Courties et al., 2015; Issa and Griffin, 2012; Messier, 2009; Rai and Sandell, 2011; Thijssen et al., 2015). Furthermore, many studies have shown that components of metabolic syndrome, including hypertension, hyperglycemia, dyslipidemia and central adiposity, are associated with OA pathology and the risk of progression (Berenbaum et al., 2017; Katz et al., 2010; Sowers et al., 2009; Zhuo et al., 2012). In particular, articular cartilage is susceptible to the deleterious effects of both elevated glucose and lipids. For example, high dietary fat consumption (Lu et al., 2016) and type 2 diabetes (Eymard et al., 2015) are each associated with more rapidly progressing joint space narrowing in individuals with knee OA, even after adjusting for BMI. However, the causal role of metabolic syndrome and its components in knee OA progression remain unclear (Appleton et al., 2017; Niu et al., 2017). Thus, experimentally isolating the effects of specific dietary macronutrients on cartilage metabolism and joint pathology might improve our understanding of how metabolic factors contribute to OA pathogenesis.

The use of diet-induced obese animal models to study OA pathogenesis has expanded rapidly in the past 10 years (Berenbaum et al., 2017; Griffin and Guilak, 2008). However, variability in the timing and severity of high-fat-diet-induced OA phenotypes, particularly in mice, has raised questions about the robustness and repeatability of the model (Kozijn et al., 2017). One potential source of variation is the comparison of defined high-fat diets to undefined chow diets, which differ in many ways other than just fat content (Warden and Fisler, 2008). For example, chow diets contain high levels of isoflavones, which can influence hormone-dependent rodent phenotypes (Brown and Setchell, 2001). Another difference between chow and defined diets is fiber type and content. Defined diets often include an insoluble fiber, cellulose, which contributes to alterations in gut morphology and microbiota content (Chassaing et al., 2015; Dalby et al., 2017). Moreover, even when defined control and high-fat diets are used, the specific composition of macronutrients can vary and contribute to metabolic perturbations. Wu and colleagues elegantly showed the importance of dietary fatty acid composition in post-traumatic OA pathogenesis (Wu et al., 2015).

An area that has received less attention in diet-induced obese animal studies of OA is carbohydrate type and content. We recently reported the time course of OA pathogenesis in male C57BL/6J mice fed a high-fat diet (60% kcal fat; ResearchDiets, D12492) compared with a defined control-fat diet (10% kcal fat; ResearchDiets, D12450B) (Barboza et al., 2017). High-fat feeding did not increase the overall knee OA score versus control diet until >32 weeks of high-fat feeding. This duration was longer than expected, based on our previous work showing early-stage OA changes after 12 weeks using the same 60% high-fat diet; however, in this earlier study, the control animals ate an undefined chow diet (Griffin et al., 2012). Thus, differences in the dietary compositions of the control diets might contribute to the variation in outcomes. In particular, it is common for many defined low-fat control diets, which often contain nearly 70% kcal from carbohydrates, to include a substantial amount of sucrose. Sucrose is a disaccharide composed of glucose and fructose. In people, fructose consumption independently causes dyslipidemia and abdominal adiposity (Warden and Fisler, 2008). Thus, diet manufacturers began producing diets that matched the sucrose content of control low-fat diets to that in the high-fat diet to control for potential effects of sucrose. We incorporated a recently developed low-fat diet with lower sucrose content to further understand how dietary sucrose independent of fat contributes to OA pathophysiology in the C57BL/6J mouse model of diet-induced OA.

We compared the independent effects of dietary sucrose and fat on knee OA pathology and cartilage metabolism by feeding C57BL/6J mice one of three defined diets: (1) 10% kcal fat diet with sucrose content matched to that contained in the high-fat diet (7%), herein defined as low fat low sucrose (LFLS); (2) the previously used 10% kcal fat diet with higher sucrose content (35%), referred to as low fat high sucrose (LFHS); and (3) the previously used high-fat diet containing 60% kcal fat and 7% kcal sucrose, referred to as high fat low sucrose (HFLS) (Table S1). The definitions of the diet groups as LFLS, LFHS and HFLS are intended to distinguish the relative sucrose and fat content between the groups, although the LFLS group is similar to chow diets in terms of sucrose and fat content. We hypothesized that increased dietary sucrose and fat (i.e. LFHS and HFLS diets, respectively) would induce systemic metabolic pathology, cartilage stress and early-stage markers of OA pathology compared with the LFLS diet group. We focused on 20 weeks of diet treatment, which causes early-stage OA pathology in the HFLS group (Barboza et al., 2017).

## RESULTS

### Dietary sucrose content in low-fat diets changes serum metabolic biomarkers without altering body weight or fat

**Fig. 1.**
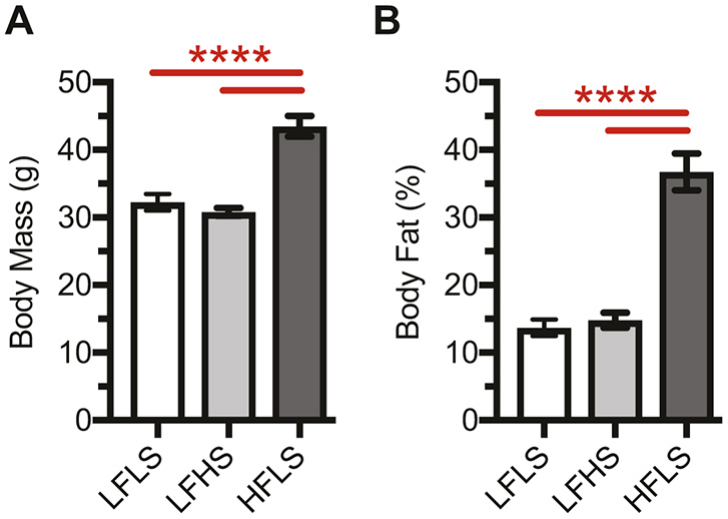
**Effect of diet on body mass and adiposity.** (A,B) Increased dietary fat content, but not sucrose, increased body mass (A) and body fat (B). Data were collected at 25 weeks of age after 19 weeks of diet treatment. Body fat, expressed as a percent of total body mass, was measured by dual-energy X-ray absorptiometry (DEXA). Data shown are for one cohort of animals, although values are consistent with data from additional cohorts. Values are mean±s.e.m. Lines over bars indicate statistically significant differences (****P<0.0001 by one-way ANOVA and Tukey's multiple comparisons test; LFLS, *n*=10; LFHS, *n*=8; HFLS, *n*=10).

High dietary sucrose content did not alter body weight or percentage body fat content in mice fed low-fat diets (i.e. 10% kcal fat, 70% kcal carbohydrate) (Fig. 1A,B, LFLS versus LFHS). In contrast, mice fed a HFLS diet gained substantially more body weight (∼40% increase) and body fat (∼2.5-fold increase) compared with low-fat fed mice, regardless of sucrose content (Fig. 1A,B). To further characterize the metabolic effects of dietary sucrose and fat content, we also analyzed a panel of diagnostic serum metabolic markers in blood collected at the time of death. Upon necropsy, we observed tumor-like nodules in the liver of two LFLS animals. The presence of nodules was associated with altered serum metabolites and synovial thickening (Fig. S1); therefore, these samples were excluded from the final serum and synovial analyses, as well as cartilage gene and protein analyses. We did not, however, observe differences in cartilage or osteophyte histopathology scores associated with liver nodules so these samples were included in those outcome analyses.

**Fig. 2.**
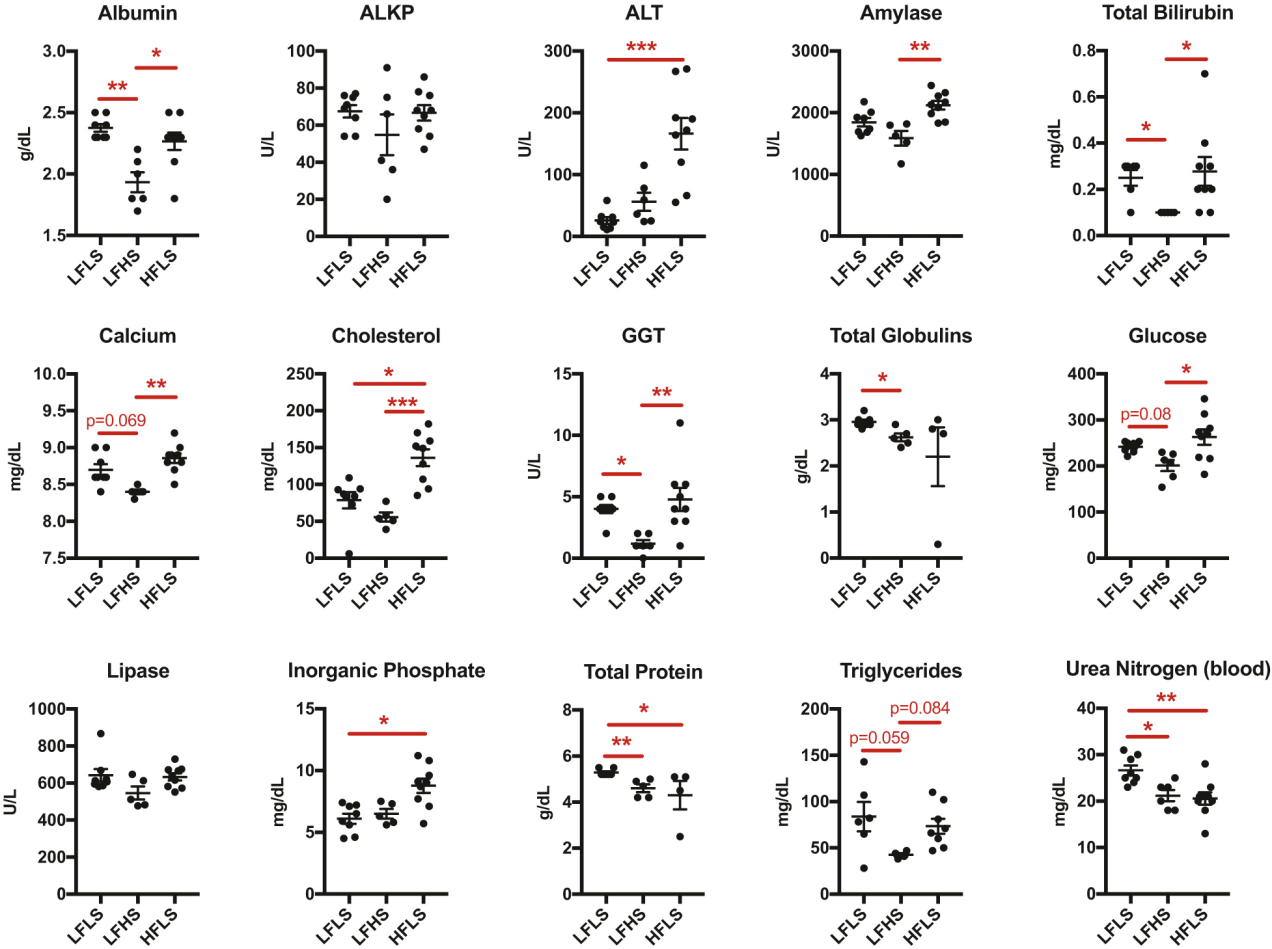
**Dietary sucrose and fat independently alter serum metabolic biomarkers.** Metabolic markers were generally lower in mice fed the LFHS diet compared with those fed the LFLS diet. Consequently, markers were more likely to be significantly elevated in mice fed a HFLS diet when compared with mice fed a low-fat diet that contained more, not less, sucrose. Blood was obtained by terminal cardiac puncture between 09:00 and 11:00 following a 1-2 h fast. Data points are values for individual animals, and horizontal bars are mean±s.e.m. Results that include two LFLS samples excluded due to liver nodules are shown in Fig. S1. Lines over bars indicate statistically significant differences (*P<0.05, **P<0.01, ***P<0.001 by one-way ANOVA and Tukey's multiple comparisons test or Kruskal–Wallis test with Dunn's multiple comparisons test; LFLS, *n*=6-8; LFHS, *n*=5-6; HFLS, *n*=4-9). Variations in sample size were due to marker detectability. ALKP, alkaline phosphatase; ALT, alanine aminotransferase; GGT, gamma-glutamyltransferase.

Overall, metabolic markers were more likely to be significantly elevated in mice fed a HFLS diet when compared with mice fed a low-fat diet that contained more, not less, sucrose (i.e. LFHS) (Fig. 2). This is because metabolic markers were generally lower in mice fed the LFHS diet compared with those fed the LFLS diet (Fig. 2). For example, serum albumin, bilirubin, gamma-glutamyltransferase, globulins, protein and urea nitrogen were all significantly reduced in LFHS versus LFLS mice. Moreover, serum calcium, glucose and triglycerides also trended lower in LFHS mice. In fact, mice fed a LFLS diet had elevated levels of total serum protein and urea nitrogen compared with mice fed any other diet, suggesting that this diet might stimulate protein catabolism.

Notably, only two metabolites were elevated in the HFLS versus LFLS diet group: alanine aminotransferase (ALT) and inorganic phosphate (Fig. 2). In addition, serum cholesterol was greater in mice fed a HFLS diet regardless of sucrose content in the low-fat diets. High dietary sucrose did not significantly elevate any metabolite levels compared with the other diets. Overall, these data show that dietary sucrose significantly alters serum markers of metabolic function independent of changes in body weight or fat. Contrary to our expectation, more serum metabolites were altered in animals fed a high-fat diet when compared with those fed a control diet with more, not less, sucrose. Consequently, interpreting the effect of high-fat-diet-induced obesity on serum metabolites depends on the carbohydrate composition of the control low-fat diet.

### Low dietary sucrose and high dietary fat induce different histological changes in knee cartilage and bone that are associated with early-stage OA

**Fig. 3.**
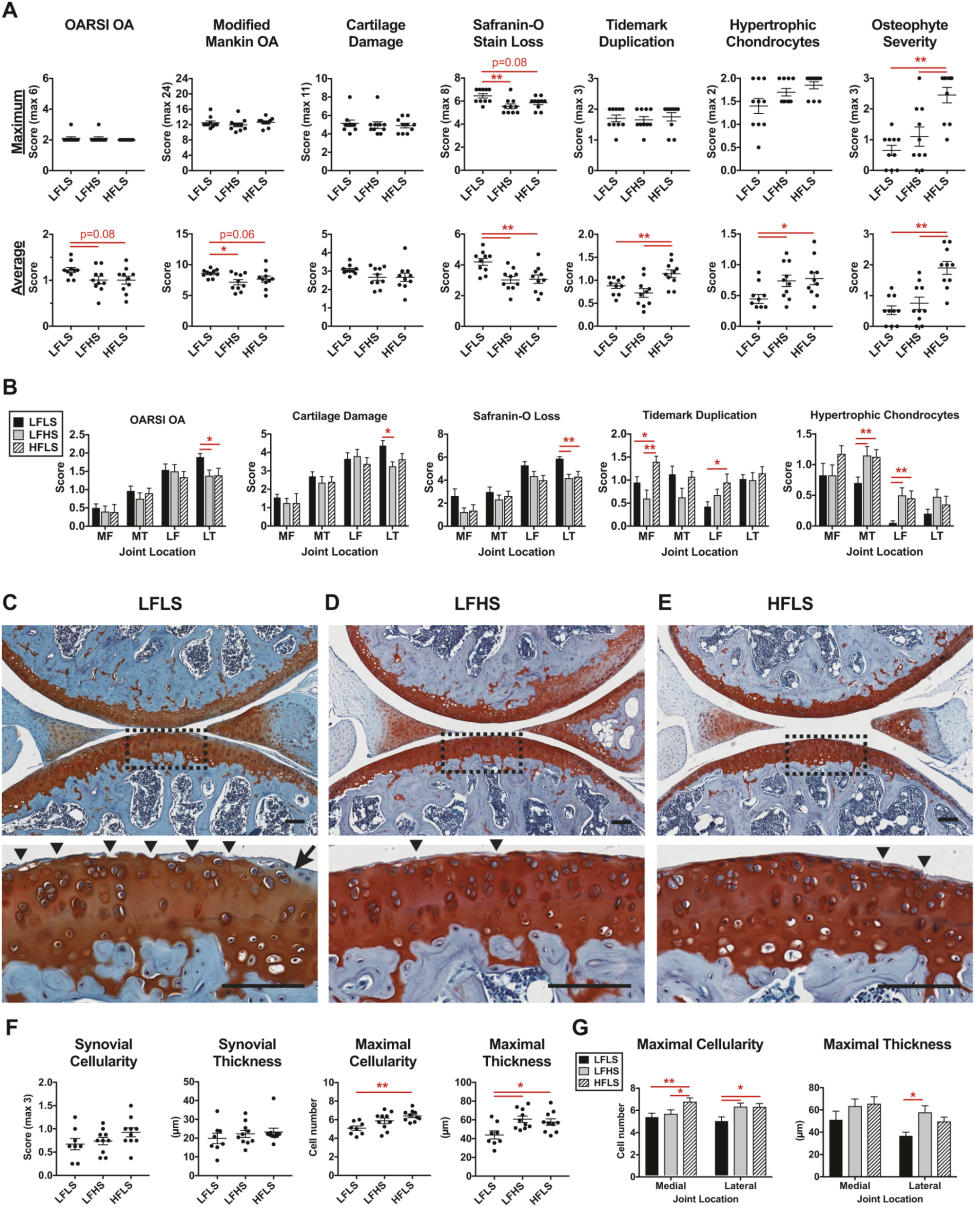
**Dietary sucrose and fat independently alter knee OA pathology.** (A) Cartilage OA histopathology scoring of medial and lateral knee compartments. Osteophyte scoring from medial tibial compartment. Data points represent values for individual animals, and horizontal bars are mean±s.e.m. (B) Location-specific OA histopathology (LF, lateral femur; LT, lateral tibia; MF, medial femur; MT, medial tibia). Data are mean±s.e.m. (C-E) Representative sagittal section histology images from the lateral knee compartment. Scale bars: 100 µm. Dashed line rectangular area from the lateral tibial plateau is shown at higher magnification in the lower image to illustrate the greater prevalence of cartilage fibrillation (arrowheads) and Safranin-O staining loss (arrow) in the LFLS group than in the LFHS and HFLS groups. (F) Representative and maximal synovial lining cellularity and thickness. (G) Maximal focal synovial cellularity and thickness by joint compartment. Lines over bars indicate statistically significant differences (*P<0.05, **P<0.01) by Kruskal–Wallis (A,F) or repeated-measures two-way ANOVA (B,G) with FDR-corrected post hoc tests (0.05). *n*=10 for each diet group, except for the LFLS group in F and G (*n*=8).

We evaluated OA histopathology scoring using two complementary approaches, the maximum joint score and the overall location-averaged joint score (Fig. 3A). The maximum score comparison is more consistent with clinical definitions of disease burden, whereas the average score is less sensitive to small focal changes and thus better represents more widespread pathological changes. We previously reported overall site-average scoring for the LFHS and HFLS joints (Barboza et al., 2017), but these histological sections were blinded and re-graded together with the LFLS sections for consistency. Based on maximum scores, the knee OA pathology was moderate and did not differ between the three diets using either the Osteoarthritis Research Society International (OARSI) or modified Mankin scoring systems (Fig. 3A; Table S2). The average whole-joint OA pathology, however, showed minor significant or trending increases for the LFLS group (Fig. 3A). The primary factor driving the slight increase in early signs of OA in the LFLS group was a greater loss of Safranin-O staining, indicating reduced proteoglycan content. These changes were greatest in the lateral tibial plateau, where superficial cartilage damage was also increased (Fig. 3B,C). In contrast, other histopathological changes were greatest in the LFHS and HFLS diet groups. Notably, a high-fat diet increased osteophyte severity and cartilage tidemark duplication (Fig. 3A,B). Moreover, when scores were averaged throughout the joint, the LFHS and HFLS groups showed increased numbers of hypertrophic chondrocytes in the calcified cartilage (Fig. 3A,B). The LFHS and HFLS groups also showed increases in the maximal synovial cellularity and thickness in focal areas adjacent to the meniscus (Fig. 3F,G; Fig. S2). Thus, animals on each diet showed different characteristics of early-stage OA pathology.

### Diet composition alters the expression of cellular stress response genes in cartilage

**Fig. 4.**
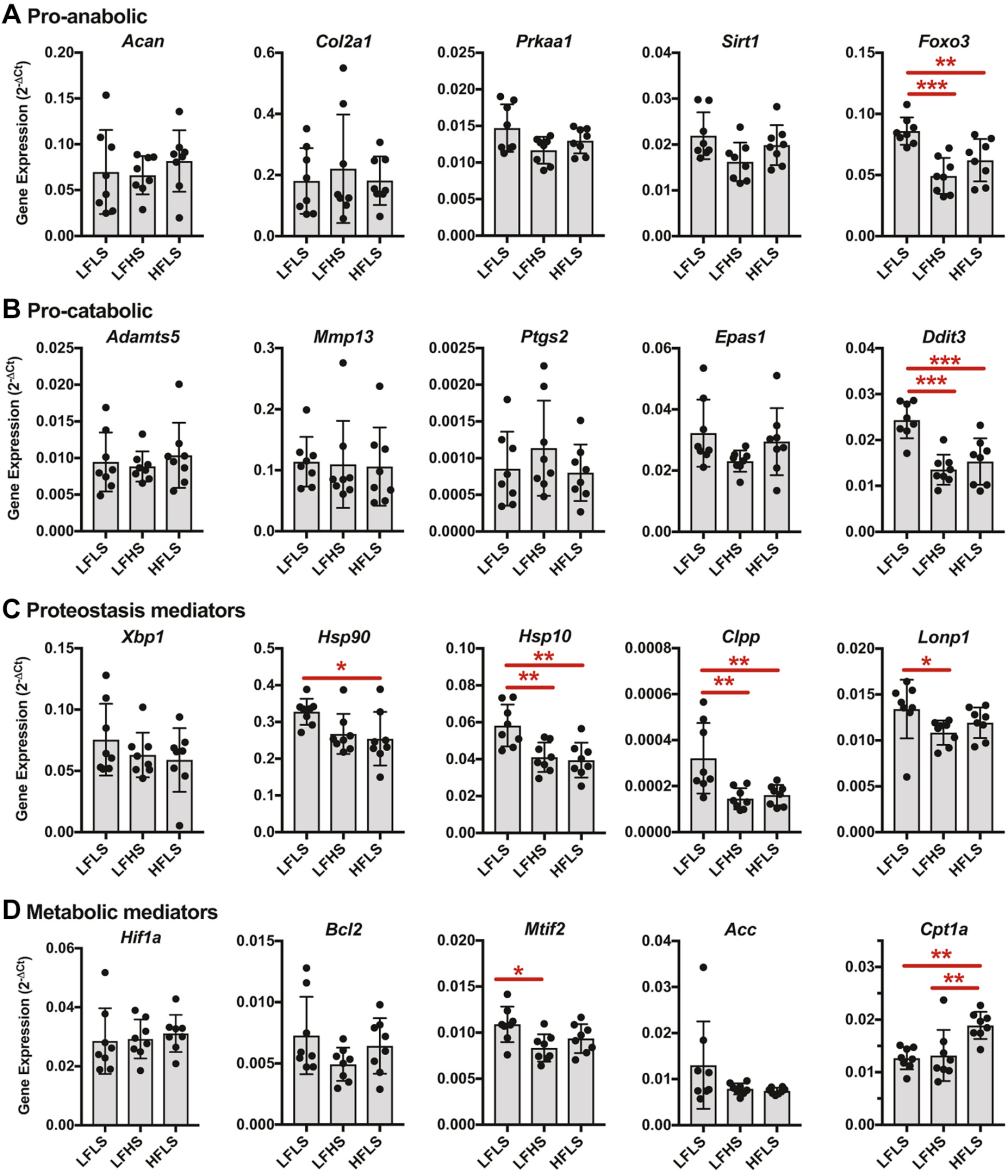
**Effects of dietary sucrose and fat on the expression of metabolic and stress-response genes in knee cartilage.** (A-D) Data are presented as expression relative to the geometric mean of three reference genes rather than as fold-change relative to the LFLS group, owing to the absence of longitudinal data for assessing diet-induced up- versus downregulation. Diet did not substantially alter the expression of cartilage anabolic genes (A) (e.g. *Acan* or *Col2a1*) or cartilage catabolic genes (B) (e.g. *Adamts5* and *Mmp13*). However, diet did alter the expression of cellular stress mediators *Foxo3*, a transcription factor that positively regulates chondrocyte autophagy and antioxidant expression, and *Ddit3* (i.e. *Chop*), a mediator of endoplasmic reticulum stress and the mitochondrial unfolded protein response. Further evaluation of genes involved in cellular proteostasis (C) and metabolism (D) showed significant effects of diet on mediators of cartilage mitochondrial proteostasis and lipid metabolism. Detailed cellular stress and metabolism gene expression data, including gene NCBI RefSeq numbers, are provided in Table S3. Lines over bars indicate statistically significant differences. *P<0.05, **P<0.01 and ***P<0.001 by one-way ANOVA and Tukey's multiple comparisons test or Kruskal–Wallis test with Dunn's multiple comparisons test; *n*=8 per diet group.

We first evaluated the expression of pro-anabolic and pro-catabolic genes in cartilage that was isolated from the femoral condyles and tibial plateau (Fig. 4A,B, respectively). The minor changes in knee joint histopathology were not associated with significant changes in the expression of primary cartilage anabolic genes (e.g. *Acan* or *Col2a1*) or catabolic genes (e.g. *Adamts5* and *Mmp13*) (Fig. 4A,B). Thus, we next evaluated the expression of genes associated with the protection against, and induction of, cellular stress. The expression of the AMP-activated protein kinase subunit *Prkaa1* and the nuclear NAD-dependent deacetylase sirtuin-1 (*Sirt1*) showed near-significant trends for differences across the diet groups (*P*=0.058 and *P*=0.063, respectively; one-way ANOVA) (Fig. 4A). These trends were primarily caused by increased expression in the LFLS versus LFHS diet groups [*P*=0.058 and *P*=0.067, respectively; false discovery rate (FDR)-adjusted post-hoc comparison]. Moreover, the expression of the transcription factor *Foxo3*, which positively regulates chondrocyte autophagy and antioxidant expression, was significantly altered by diet (*P*=0.0002; one-way ANOVA). Like *Prkaa1* and *Sirt1*, *Foxo3* was also most highly expressed in the LFLS group (Fig. 4A). Diet did not alter the expression of genes transcribing the cellular stress mediators PGE2 and HIF-2α (*Ptgs2* and *Epas1*, respectively; Fig. 4B). However, diet did alter the expression of the endoplasmic reticulum (ER) stress mediator *Ddit3*, also known as *Chop*, with expression greatest in the LFLS group (*P*<0.0001, one-way ANOVA; Fig. 4B). CHOP is a transcription factor for which expression is upregulated in response to ER stress and the mitochondrial unfolded protein response (mtUPR) (Shpilka and Haynes, 2017).

To further characterize these diet-dependent cellular stress responses, we next analyzed the expression of genes involved in cellular protein homeostasis (i.e. ‘proteostasis’) and metabolism. These findings suggest that dietary sucrose and fat content led to differences in cartilage mitochondrial proteostasis and lipid metabolism. Diet did not significantly alter the expression of the transcription factor X box-binding protein 1 (*Xbp1*), a key regulator of the ER stress response (Fig. 4C); however, it did alter the expression of the ubiquitous adenosine triphosphate (ATP)-dependent molecular chaperone *Hsp90* (also known as *Hsp90aa1*) and the mitochondrial-localized chaperone *Hsp10* (also known as *Hspe1*). In both cases, expression was greatest in cartilage from the LFLS diet group (Fig. 4C). Furthermore, diet also altered the expression of the mitochondrial protease subunits *Clpp* and *Lonp1*, which were also most highly expressed in the LFLS diet group (*P*=0.0012 and *P*=0.09, respectively; one-way ANOVA). There were no differences in the expression of *Hif1a* or the autophagy mediator *Bcl2* across diets, although a key regulator of mitochondrial translation, *Mtif2*, was more highly expressed in the LFLS versus LFHS group (Fig. 4D).

Based on the potential role of diet-induced changes in cartilage lipid metabolism, we also evaluated the expression of two important lipid metabolism regulatory enzymes: acetyl-CoA carboxylase (*Acc*) and carnitine palmitoyltransferase 1 (*Cpt1a*). *Acc* activity inhibits the beta-oxidation of fatty acids and promotes lipid synthesis; whereas, *Cpt1a* shuttles fatty acids into the mitochondria and is the rate-limiting step of beta-oxidation. Diet did not alter *Acc* expression, although variation was much greater in the LFLS group compared with the LFHS or HFLS groups. In contrast, a high-fat diet significantly increased *Cpt1a* expression regardless of the sucrose content of the low-fat diets, suggesting a potential increase in cartilage beta-oxidation (Fig. 4D). Detailed cellular stress and metabolism gene expression data are provided in Table S3.

### High dietary sucrose is associated with lower levels of cartilage metabolic and antioxidant proteins compared with high dietary fat

**Fig. 5.**
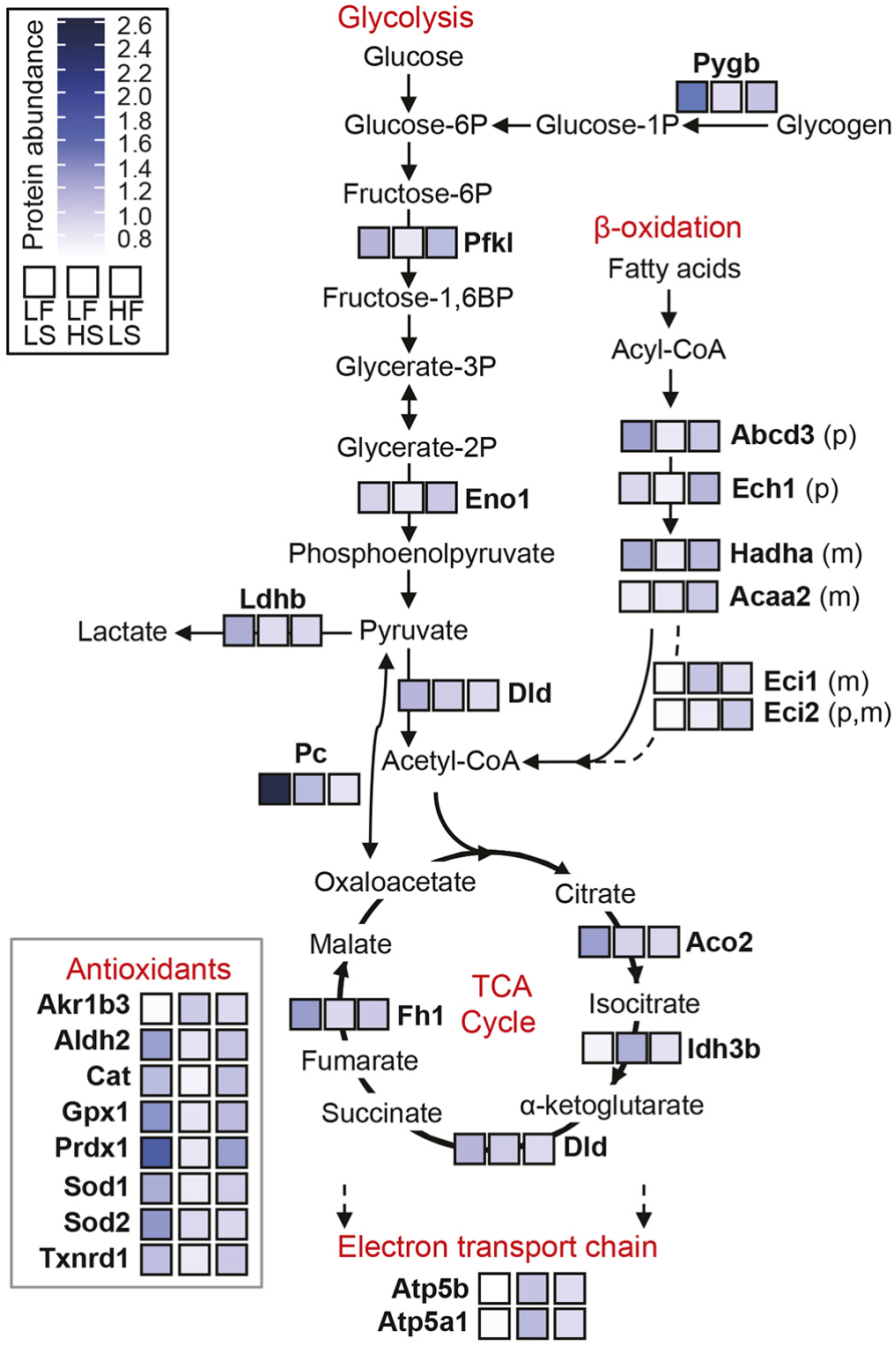
**Effects of dietary sucrose and fat on changes in metabolic and antioxidant enzyme abundance in knee cartilage.** Heatmap of protein abundance levels as measured by selected-reaction-monitoring (SRM) mass spectrometry. Enzymes with a significant diet effect (one-way ANOVA; *P*<0.05) are shown, with relative abundance indicated by color scale. More diet-induced differences occurred with elevated dietary sucrose (20 proteins; LFHS versus LFLS) compared with dietary fat (7 proteins; HFLS versus LFLS). These differences were generally caused by protein levels being lower in the LFHS versus LFLS group, with HFLS group levels being intermediate. When comparing elevated dietary fat versus sucrose (i.e. HFLS versus LFHS), high dietary fat was associated with greater levels of proteins involved in glycolysis (Pfkl, Eno1), β-oxidation (Ech1, Hadha, Acaa2), and antioxidant defense (Gpx1). For β-oxidation enzymes, ‘p’ indicates localization to the peroxisome and ‘m’ indicates localization to mitochondria. Protein names and diet-specific differences based on Tukey post hoc statistical analysis are provided in Table S4. TCA, tricarboxylic acid.

We next utilized selected-reaction-monitoring (SRM) mass spectrometry to quantify protein abundance in knee cartilage homogenates. The analysis targeted 120 proteins spanning the glycolysis, beta-oxidation, tricarboxylic acid (TCA) cycle and electron transport chain pathways, as well as enzymes involved in cellular antioxidant defense and proteostasis. Of these, 100 proteins were sufficiently abundant for quantification. A list of all detected proteins and their diet-specific differences in abundance is provided in Table S4. Twenty-seven proteins were significantly altered by diet. These proteins were distributed throughout all the metabolic and antioxidant pathways that were evaluated (Fig. 5).

Overall, the greatest number of diet-induced differences occurred with elevated dietary sucrose (20 proteins; LFHS versus LFLS) compared with dietary fat (7 proteins; HFLS versus LFLS). These differences were generally due to protein levels being lower in the LFHS versus LFLS group, with HFLS group levels being intermediate. For example, the proteins that were less abundant with increased dietary sucrose included nearly all of the antioxidant proteins and many involved in glycolysis [e.g. glycogen phosphorylase (Pygb), phosphofructokinase (Pfkl), lactate dehydrogenase (Ldhb)] and the TCA cycle [e.g. aconitase (Aco2), fumarate hydratase (Fh1), pyruvate carboxylase (Pc)] (Fig. 5). There were some notable exceptions, however, where protein abundance was increased with high dietary sucrose. These proteins include the TCA cycle enzyme isocitrate dehydrogenase (Idh3b), complex V of the electron transport chain [i.e. mitochondrial ATP synthase (Atp5b, Atp5a1)], and aldo-keto reductase family 1 member B3 (Akr1b3), which is the first and rate-limiting enzyme in the polyol pathway (Fig. 5).

As previously mentioned, there were fewer differences in protein abundance associated with high dietary fat (i.e. HFLS versus LFLS). Proteins that were uniquely elevated in the HFLS group involve those that regulate the beta-oxidation of fatty acids, including acetyl-coenzyme A acyltransferase 2 (Acaa2) and enoyl-coenzyme A delta isomerase (Eci2). More proteins, however, were lower in the HFLS group, including Ldhb, Aco2, Pc, superoxide dismutase 2 (Sod2), and dihydrolipoamide dehydrogenase (DLD), an E3 component of pyruvate and α-ketoglutarate dehydrogenases. When comparing elevated dietary fat versus sucrose (i.e. HFLS versus LFHS), high dietary fat was associated with greater levels of proteins involved in glycolysis (Pfkl, Eno1), beta-oxidation (Ech1, Hadha, Acaa2), and antioxidant defense (Gpx1) (Fig. 5; Table S4).

### High dietary fat greatly reduces the network connectivity of cartilage metabolic and cellular stress proteins

**Fig. 6.**
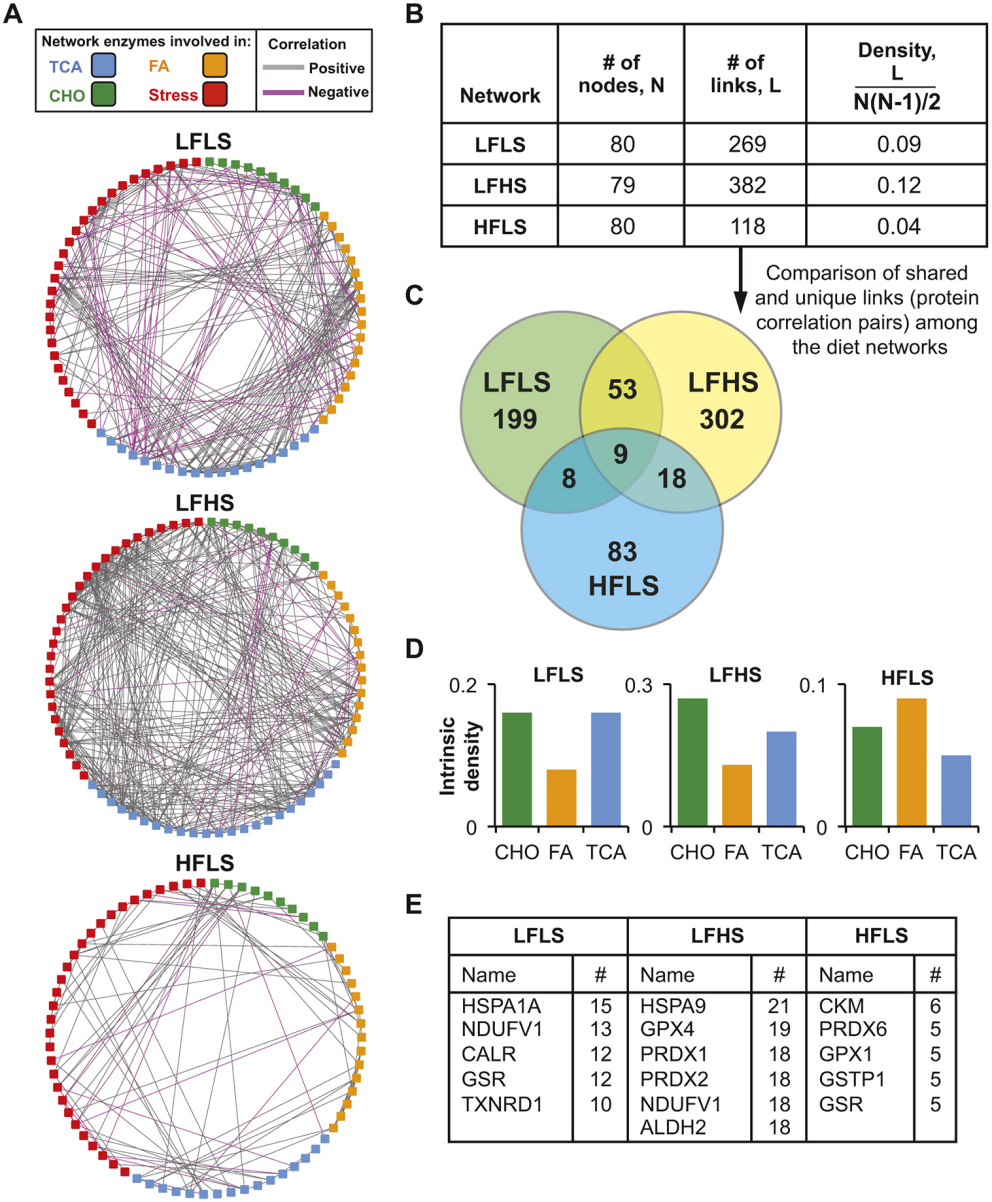
**Correlation-based network analysis of cartilage metabolic and stress-response antioxidant enzymes following high-sucrose and high-fat diets.** Networks were developed based on significantly correlated protein pairs (i.e. correlation coefficient |*r*|≥0.8, *P*≤0.05) among independent diet-specific cartilage samples analyzed by SRM mass spectrometry. (A) Graphical network representation for each diet. Squares represent one protein (i.e. ‘node’) with at least one significant correlation (i.e. ‘link’) to another protein. Enzyme functional categories and direction of correlation are indicated by the key. (B) Network density calculations show substantial diet effects, being greatest with a high-sucrose diet and least with a high-fat diet. (C) Venn diagram of the correlated enzyme pairs shows that most correlations are diet specific. (D) Network densities intrinsic to specific metabolic pathways. Carbohydrate metabolism and TCA cycle pathway densities were elevated for LFLS and LFHS diets, whereas fatty acid metabolism density was relatively greatest with a HFLS diet. (E) Diet-specific comparison of proteins with the greatest number of links to other proteins (i.e. ‘hub’ proteins). Full protein names are available in Table S4.

The analysis of metabolic and antioxidant protein levels in cartilage from LFLS, LFHS and HFLS diet groups showed differences across multiple pathways, suggesting substantial effects of dietary sucrose and fat content on cartilage metabolic regulation. Therefore, we sought an integrative analytical approach to evaluate these effects using a correlation-based network analysis. Correlation network analyses, also referred to as co-expression analyses, can provide inference into biological function when quantitative relationships between pairs of values, in this case protein levels, are considered in aggregate. For our analysis, we evaluated each diet separately to define a diet-specific protein network (Fig. 6A). These networks were based on protein pairs with significant correlations among independent within-diet sample replicates (i.e. correlation coefficient |*r*|≥0.8 and *P*≤0.05). We focused on differences in overall network size and density (Fig. 6A-C), metabolic pathway-specific intrinsic network density (Fig. 6D) and ‘hub’ proteins with the greatest number of correlations (Fig. 6E).

We observed substantial differences in network topology among the three diets (Fig. 6A). The diet effects were almost exclusively caused by differences in the number of correlations among protein pairs (i.e. the number of ‘links’) rather than differences in the number of proteins with ≥1 significant correlation (i.e. ‘number of nodes’), which was nearly identical among diets (Fig. 6B). Consequently, the network density varied considerably among diets, being greatest in the LFHS diet (0.12) and least in the HFLS diet (0.04) (Fig. 6B). Notably, the LFLS diet network was intermediate to both diets and contained a similar number of positive and negative correlations (Fig. 6A,B). Of >750 protein pair correlations among all three diets, only nine were common to all three diets (Fig. 6C). In general, the majority of protein pair correlations were unique to each diet. We next evaluated the network densities that were intrinsic to specific metabolic pathways. These results showed that network densities of enzymes involved in carbohydrate metabolism and the TCA cycle were relatively greatest in the LFLS and LFHS diets, whereas fatty acid metabolism enzyme densities were relatively greatest in the HFLS diet (Fig. 6D). Finally, we compared the proteins with the greatest number of significant correlations with other proteins within each diet network (i.e. ‘hub’ proteins) (Fig. 6E). This comparison showed that stress-related proteins (i.e. antioxidant and proteostasis proteins) were the most common hub proteins in each network. However, the specific proteins mostly differed between the diets, and the number of correlations associated with the top-ranked proteins reflected the diet-specific network density differences (i.e. greatest in LFHS and least in HFLS) (Fig. 6E).

## DISCUSSION

We hypothesized that increased dietary sucrose and fat would induce systemic metabolic pathology, cartilage stress, and early-stage markers of OA pathology compared with the LFLS diet group. Our findings showed broad effects of dietary sucrose and fat in all of these outcomes, as summarized in Table 1. Notably, the greatest number of changes occurred between the low- and high-sucrose diets despite no differences in body mass or body fat. For example, there were more than twice as many differences in cartilage gene expression and protein abundance between the low- and high-sucrose diets compared with the low- and high-fat diets (Table 1). However, contrary to our hypothesis, more pathologic changes were associated with a low-sucrose diet. For example, cartilage pathology and serum metabolic disease biomarkers were reduced in the high-sucrose diet group (Table 1). Consequently, for studies using high-fat diets to study obesity-induced OA, the carbohydrate composition of the low-fat control diet has the potential to significantly alter the interpretation of obesity-related outcomes (e.g. compare the ‘Increased fat’ and ‘Mixed comparison’ column summaries in Table 1).

**Table 1.**
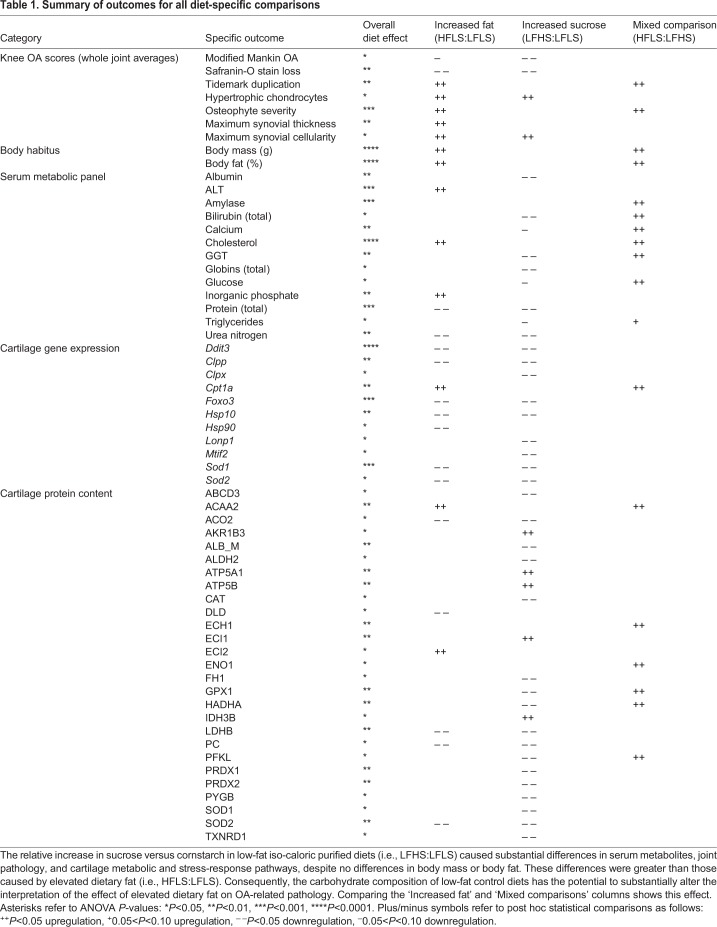
**Summary of outcomes for all diet-specific comparisons**

The importance of the composition of control diets in high-fat-diet-induced obesity studies of OA pathophysiology has not been previously recognized. In fact, we recently reviewed how the variation in high-fat diet content and composition between studies is a potential reason for differences in OA outcomes (Berenbaum et al., 2017). However, based on our current findings, variation in control diets could also be a contributing factor. We compiled a follow-up to Table 2 of Berenbaum et al. (2017), which includes the control diets used in studies of diet-induced obesity and OA (Table S5). This table shows that like the variation in high-fat diets, there is also substantial variation among control diets, including the use of nonpurified ‘chow’ diets versus purified low-fat diets. This added variation in control diet composition could further contribute to differences reported in the literature (Kozijn et al., 2017).

What is the ideal control diet for diet-induced obese mouse studies of OA? From a macro-nutrient perspective (i.e. % kcal from protein, carbohydrates and fat), both the LFLS and LFHS diets are similar to many chow diets. However, the lower sucrose content of the LFLS diet is more similar to chow, which typically contains little to no sucrose. The LFLS diet is also the most appropriate control diet for comparison to the HFLS diet because the only differences are the relative content of cornstarch versus lard. However, the use of cellulose as an insoluble fiber supplement in many purified defined diets, such as those used in the current study, causes substantial changes in gut morphology and microbiota content compared with chow-fed mice (Chassaing et al., 2015; Dalby et al., 2017). These changes could underlie our findings indicating that the LFLS diet was moderately stressful to the mice. Numerous serum metabolic biomarkers were elevated in the LFLS diet group, including serum albumin, bilirubin, gamma-glutamyltransferase, globulins, protein and urea nitrogen. These findings suggest that liver function was impaired with the LFLS diet and that protein catabolism might have been increased. In addition, two animals developed tumor-like liver nodules. A direct comparison to chow-fed mice would be needed to test this idea, although the comparison to LFHS-fed mice suggests a potential benefit of sucrose.

It is not known how increased sucrose content improved metabolic health, although one way could be by increasing palatability. A recent dietary preference study in rats using the same diets as the current study showed a strong preference for the HFLS diet and a secondary preference for the LFHS diet, with the relative consumption being 4%, 10% and 86% of total calories for the LFLS, LFHS, and HFLS diets, respectively (Lee et al., 2017). Thus, palatability could contribute to differences among diets, perhaps by reducing stress (Finger et al., 2011). An additional factor that could be modified to improve the health of mice fed purified LFLS diets is by changing the composition of the fiber supplement. Swapping insoluble cellulose for a soluble fiber, inulin, has been shown to protect against the loss of gut mass and recover fecal short-chain fatty acid levels close to those observed in chow-fed mice (Chassaing et al., 2015). Recent studies have linked altered gut microbiota to OA pathology (Collins et al., 2015; Schott et al., 2018), suggesting that the fiber composition of purified, defined diets is an important consideration in diet selection. Thus, although the current study does not clearly identify the appropriate ‘control’ diet for diet-induced obese mouse studies of OA, it shows that controlling for body weight or body fat alone is insufficient. Future studies are needed that include longitudinal testing and the modification of purified, defined low-fat diets to improve palatability and fiber composition.

Our findings shed light on an important question within the field, which is to what extent do changes in systemic metabolism alter joint tissue metabolism? In terms of systemic factors, we observed that elevated levels of total serum protein and urea nitrogen, factors potentially linked to increased protein catabolism or kidney or liver disease, were associated with reduced cartilage Safranin-O staining in LFLS-fed mice. Low-grade systemic inflammation and hypertension are both linked to OA progression clinically (Attur et al., 2015; Niu et al., 2017), although they were not tested in the current study. In HFLS-fed mice, elevated cholesterol was associated with increased cartilage tidemark duplication and osteophyte formation. High cholesterol has previously been shown to increase ectopic bone formation in experimental mouse studies (de Munter et al., 2013), and dysregulated cholesterol levels and elevated dietary fat have been associated with OA in humans (Berenbaum et al., 2017; Clockaerts et al., 2011; Lu et al., 2016).

In cartilage, we observed changes in gene expression and protein abundance, consistent with distinct diet effects on substrate metabolism. For example, our findings suggest that a high-fat diet increases cartilage lipid metabolism. The HFLS diet increased *Cpt1a* expression, which encodes the enzyme mediating the rate-limiting step of beta-oxidation and has been implicated in a role for the nuclear receptor peroxisome proliferator-activated receptor δ as a promoter of cartilage degeneration (Ratneswaran et al., 2015, 2017). The HFLS diet also increased the terminal enzyme of beta-oxidation, acetyl-coenzyme A acyltransferase 2. Furthermore, a pathway-specific network analysis of carbohydrate versus fatty acid metabolic pathways showed that the fatty acid network density was elevated with the HFLS diet. Conversely, an increase in dietary sucrose modestly increased the carbohydrate network density relative to the LFLS group. In addition, the increase in Akr1b3 in the LFHS group suggests that sucrose increased the polyol metabolic pathway, which was also observed in primary chondrocytes cultured in high glucose (Laiguillon et al., 2015). The consequences of increased polyol pathway flux are not well understood in chondrocytes, but they could involve altered cellular redox homeostasis and enhanced chondrocyte inflammation (Laiguillon et al., 2015).

We previously reported that high-fat-diet-induced obesity did not increase overt OA cartilage pathology until >32 weeks of diet treatment using the HFLS versus the LFHS diets (Barboza et al., 2017). Therefore, an additional goal of this study was to identify early molecular changes in cartilage that might contribute to the future increase in cartilage pathology. There were few differences in cartilage gene expression or protein abundance in the HFLS versus LFHS groups. The most notable differences were in genes and proteins associated with elevated lipid metabolism. However, a correlation network analysis of metabolic and stress-response proteins revealed stark differences between these two diets in terms of the density of the networks and the specific pairs of correlated proteins (i.e. ‘links’). The HFLS network density was only one-third that of the LFHS diet, despite a similar-sized network. Changes in network density have previously been associated with different types and durations of cellular stress responses. In yeast, oxidative stress induces denser and more tightly co-regulated gene and protein co-expression networks (Lehtinen et al., 2013). In contrast, chronic or pathologic stress conditions can lead to a reduction in network density (Szalay et al., 2007). In both cases, these changes in network density might be adaptive or reflect different temporal phases of stress responses. Therefore, it is not possible to determine from our data whether the differences in network densities between HFLS and LFHS groups reflect different adaptive or temporal readjustments to cellular stress. However, the low density of the HFLS network leaves it more susceptible to collapse. Furthermore, the stress-responsive 70 kDa heat shock proteins HSPA1A and HSPA9 were the most highly linked ‘hub’ proteins in the LFLS and LFHS diets, respectively, whereas the HFLS network did not contain any highly linked heat shock proteins.

This study has several limitations. We found that numerous stress-related genes and proteins were elevated in the cartilage from the LFLS group. However, with our study design, it is not possible to interpret whether gene expression is elevated in the LFLS group or suppressed in the LFHS and HFLS groups without data from earlier time points to establish a basal level of expression. Interpretation is also hindered from a functional perspective because stress-responsive genes are often initially upregulated in a homeostatic response. These limitations also apply to the interpretation of the network analyses. Future longitudinal studies are needed to establish the causal relationship of these diet treatments.

Our results do not directly indicate the molecular etiology of OA pathologies observed among the different diet groups. For example, there were no differences in gene expression of matrix components (*Acan*, *Col2a1*) or matrix degrading proteases (*Adamts5*, *Mmp9*, *Mmp13*), although gene expression might not reflect differences in protein abundance or activity. It is also possible that diet treatments, which were started prior to skeletal maturation, altered joint tissue structure by impairing matrix synthesis anabolic pathways. For example, the reduced Safranin-O staining observed in the LFLS diet group was associated with a significant increase in glycogen phosphorylase (Pygb), the enzyme that mediates glycogenolysis. This might indicate that glucose was limiting, which could affect monosaccharide supply for proteoglycan synthesis. Although we did not independently measure proteoglycan content of cartilage to verify the Safranin-O staining results, our proteomic results are consistent with them. We found that the cartilage albumin content was significantly increased in the LFLS group (Table S4, ALB_M), and albumin content in cartilage has been shown to be inversely proportional to proteoglycan content owing to changes in the cartilage solute diffusion partition coefficient (Snowden and Maroudas, 1976). Other limitations are that we did not evaluate other OA-related joint tissue changes, such as muscle weakness, subchondral bone sclerosis, intra-articular fat inflammation, and meniscus or ligament degradation.

An additional limitation is that the diet treatments differed in the relative period of time that animals were housed at The Jackson Laboratory versus the Oklahoma Medical Research Foundation (OMRF). LFHS and HFLS animals began diet treatments at 6 weeks of age in The Jackson Laboratory's Diet-Induced Obese Mouse service before being purchased at 23-24 weeks of age. These animals were therefore only housed at the OMRF for up to 3 weeks prior to tissue collection. In contrast, the LFLS diet is not available through The Jackson Laboratory's Diet-Induced Obese Mouse service. Therefore, animals were purchased from The Jackson Laboratory at 5 weeks of age and fed the LFLS diet at the OMRF from 6 to 26 weeks of age. We did, however, attempt to minimize other potential sources of variation by evaluating joint histopathology in a blinded fashion for all groups at the same time. We also conducted all serum and gene expression analyses for all groups at the same time, and the proteomic analyses were completed in two batches that involved mixed diet group samples.

### Conclusions

Obesity is a primary risk factor for knee OA, but how pro-obesogenic diets and their various macronutrient components affect chondrocyte pathophysiology is not well understood. The results from this study show that dietary sucrose and fat content have independent effects on serum metabolic biomarkers and knee joint pathophysiology, including significant changes in cartilage metabolic and stress-responsive genes and proteins. The relative content of sucrose versus cornstarch in low-fat iso-caloric purified diets caused substantial differences in serum metabolites, joint pathology, and cartilage metabolic and stress-response pathways, despite no differences in body mass or body fat. These results illustrate the impact of metabolic factors on OA pathophysiology independent of changes in adiposity. The findings also indicate that the choice of control diets in mouse diet-induced obesity studies of OA should be carefully considered in study design and the interpretation of results. Finally, our findings suggest that increased dietary fat induces a metabolic shift in cartilage metabolism towards increased utilization of fatty acids. Thus, altered cartilage metabolism might be a contributing factor to how obesity increases the risk of OA.

## MATERIALS AND METHODS

### Animals

All experiments were conducted in accordance with protocols approved by the Association for Assessment and Accreditation of Laboratory Animal Care-accredited Institutional Animal Care and Use Committee at the OMRF. Male C57BL/6J mice were purchased from The Jackson Laboratory (USA). Beginning at 6 weeks of age, animals were fed one of three irradiated, purified open source diets (Research Diets Inc., USA; Table S1): (1) low-fat low-sucrose (LFLS) diet containing 10% kcal fat and 7% sucrose (product D12450Ji); (2) low-fat high-sucrose (LFHS) diet containing 10% kcal fat and 35% sucrose (product D12450Bi); or (3) high-fat low-sucrose (HFLS) diet containing 60% kcal fat and 7% sucrose (product D12492i). Diets were provided *ad libitum* for 20 weeks, and animals were euthanized by either CO_2_ asphyxiation or exsanguination under isoflurane anesthesia at 26 weeks of age. LFHS and HFLS animals were purchased at 23-24 weeks of age as part of The Jackson Laboratory's Diet-Induced Obese Mouse service, which randomizes animal to diet groups. Thus, these animals were acclimated to the OMRF vivarium for up to 3 weeks prior to serum and tissue collection. Animals receiving the LFLS diet were shipped at 5 weeks of age and underwent diet treatment from 6 to 26 weeks of age in the OMRF vivarium. Mice were group housed (≤5 animals/cage) in ventilated cages in a temperature-controlled room maintained at 22±3°C on 14/10 h light/dark cycles with *ad libitum* access to food and water. Animals were weighed weekly and received daily inspection and routine veterinary assessment. Body composition, excluding the head, was measured under isoflurane anesthesia using a DEXA system at 25 weeks of age (Lunar PIXImus2, GE LUNAR Corp., USA).

### Serum metabolite analysis

Animals were placed in transport cages and moved to the laboratory for a period of 1-2 h prior to blood collection between 09:00 and 11:00. We collected blood as a terminal procedure by cardiac puncture under isoflurane anesthesia. Blood was allowed to clot in microvette tubes (CB 300 Z, SARSTEDT, Germany) at room temperature for 20 min and then centrifuged at 10,000 ***g*** for 5 min. Serum was aliquoted and frozen at −80°C until analysis. Serum metabolites were measured in samples with ≥200 µl serum using an IDEXX Catalyst Dx Chemistry Analyzer (USA) with Chem 17, triglycerides and fructosamine panels following the manufacturer’s instructions.

### Histopathology analysis

Following death, the left limb was skinned and the knee was isolated by manual dissection mid-femur to mid-tibia. Periarticular skeletal muscle was retained to facilitate fixation at a physiologic joint angle, and a small incision was made near the origin of the patellar ligament to expose the joint cavity. Joints were then placed in 4% paraformaldehyde for 24 h at 4°C for tissue fixation. Following fixation, periarticular muscles were removed by gross dissection, knees were rinsed in phosphate buffered saline, and joints were decalcified using CalEx HCl-based decalcifying solution (Thermo Fisher Scientific, USA) for 3 days at 4°C. Knees were then dehydrated in an ethanol gradient prior to paraffin embedding and sagittal sectioning. Slides were stained with Hematoxylin, Fast Green and Safranin-O for histological grading as described previously (Cai et al., 2014; Griffin et al., 2010).

Two experienced graders (E.B.P.L. and T.M.G.) evaluated multiple stained sections from the medial and lateral joint compartments. Slides were organized by knee joint sample, randomized by diet treatment, and assigned a temporary identification code to blind graders to diet treatment and minimize any order effect. Modified Mankin grading and OARSI mouse OA grading scores were assigned separately for the medial tibia, medial femur, lateral tibia and lateral femur independently by each grader (Table S2). Graders also evaluated osteophyte severity semiquantitatively as previously described (Barboza et al., 2017). Subcomponent scores that differed by >2 (modified Mankin cartilage damage and Safranin-O loss) or >1 (all other scores) between graders were re-evaluated for consensus scoring. Scores were then averaged for both graders to obtain a final score per section and location. OA severity was evaluated using two complementary approaches: the maximum joint score and the overall location-averaged joint score. The maximum score is the highest score per joint for any section or joint location; this approach is more consistent with clinical definitions of disease. In contrast, the average score is less sensitive to small focal changes and thus better represents more widespread pathological changes. We previously reported overall site-average OA scoring for the LFHS and HFLS joints (Barboza et al., 2017); however, these joint sections were re-blinded and integrated with the LFLS sections to be graded again to prevent any grader-dependent discrepancies. Importantly, the previously reported findings were replicated here, indicating reproducibility of the evaluation process. Synovial cellularity and thickness was evaluated in the medial and lateral joint compartments superior and inferior to the anterior horn of the meniscus. Quantitative and semiquantitative data were collected in a blinded fashion using methods modified from Lewis and colleagues (Lewis et al., 2011) and described in detail in Fig. S2 and Table S2.

### RNA and protein extraction

Articular cartilage from the right knee was carefully collected with the aid of a stereomicroscope immediately following animal death. Femur and tibia were gently disarticulated, and meniscus and connective tissues were carefully removed from the articular surfaces using McPherson-Vannus scissors and fine forceps. Then, with a fresh size-11 scalpel blade held initially at a 45-60° angle to the cartilage surface, the blade was inserted into the cartilage and rotated almost parallel to the surface to remove cartilage without disturbing the subchondral bone. Cartilage pieces were placed in 250 µl TRIzol™ Reagent (Invitrogen, USA) on ice, flash-frozen in liquid nitrogen, and stored at −80°C until further processing. Histological evaluation of joints after cartilage removal indicates that the process captures uncalcified and calcified cartilage (data not shown). Thawed samples were mechanically homogenized using a microcentrifuge tube homogenizing pestle and rotary motor (RAE Corporation, USA) system. Samples were homogenized 3×30 s, placing samples on ice in between to minimize heating. Total TRIzol™ volume was then increased to 500 µl, and 100 µl chloroform was added before centrifuging samples for 15 min at 12,000 ***g*** at 4°C. The aqueous layer containing the chondrocyte RNA was then collected into equal volume ethanol and further processed using RNA clean and Concentrator Columns per manufacturer protocol (Zymo Research, USA). RNA was eluted in 10 μl DNase/RNase-Free Water. RNA concentration and purity were determined using a NanoDrop spectrophotometer (Thermo Fisher Scientific). Total RNA yields are typically ∼1 µg with 260/280 ratios between 1.87 and 2.05. Protein was collected from the lower organic phase fraction remaining from the TRIzol™ extraction according to the manufacturer’s protocol and processed as previously described for mass spectrometry analysis (Fu et al., 2016).

### Gene expression analysis by quantitative reverse transcription PCR array

We designed custom quantitative PCR arrays (Qiagen, USA) containing 42 target genes related to OA pathology, cellular metabolism and cellular stress (Table S3). The array included three reference genes (*B2m*, *Gapdh* and *Actb*), a positive PCR control, a positive reverse transcription control, and a negative genomic DNA control. Then, 200 ng cDNA was synthesized using the RT^2^ First-Strand kit (Qiagen) and loaded onto plates following the manufacturer’s instructions. Plates were run on a CFX96 thermocycler (Bio-Rad, USA). Data for each target gene were normalized to the geometric mean of the three reference genes, and gene expression was evaluated using the 2-*Δ*C_t_ method (Schmittgen and Livak, 2008).

### Protein abundance analysis by SRM mass spectrometry

Protein abundance was quantified using SRM mass spectrometry, as previously reported (Rindler et al., 2013). Cartilage protein (20 μg) was suspended in 1% sodium dodecyl sulfate (SDS), with 8 pmol bovine serum albumin (BSA) added as an internal standard. Samples were heated to equilibrate and proteins were precipitated with acetone. Dried protein pellets were reconstituted in 23 µl Laemmli sample buffer, and the entire sample was run into a short (1.5 cm) SDS-polyacrylamide gel electrophoresis gel. Each sample was cut from the gel as the entire lane and divided into smaller pieces. Gel pieces were washed to remove Coomassie Blue staining and then reduced, alkylated and digested overnight with trypsin. Peptide mixtures were extracted from the gel, evaporated to dryness in a SpeedVac, and reconstituted in 150 µl 1% acetic acid for analysis on a TSQ Vantage triple quadrupole mass spectrometry system (Thermo Fisher Scientific). The high-performance liquid chromatography system used was an Eksigent splitless nanoflow system with a 10 cm×75 µm inner diameter C18 reversed-phase capillary column. Aliquots of 7 µl were injected and the peptides eluted with a 60 min gradient of acetonitrile in 0.1% formic acid. The mass spectrometer was operated in the SRM mode. For each protein, a method was developed to measure two ideal peptides. Assays for multiple proteins were bundled together in larger panels. Data were analyzed using the program SkyLine to determine the integrated peak area of the appropriate chromatographic peaks. The response for each protein was calculated as the geometric mean of the area of the two protein-specific peptides. Values were then normalized to the BSA internal standard and a stable cellular reference protein, MDH1. Samples were analyzed in two separate runs [Run 1: LFHS (*n*=3) and HFLS (*n*=5); Run 2: LFLS (*n*=8), LFHS (*n*=5) and HFLS (*n*=3)], and data were normalized to the median response of all LFHS and HFLS samples for each run to minimize batch effects. The integrated data were used for statistical comparison among the three diet groups and for constructing the protein network analysis. Of 138 proteins included in the SRM analysis panels, 101 were identified in mouse articular knee cartilage (Table S4).

### Protein correlation network analysis

Protein correlation network construction and analysis was performed as previously described (Batushansky et al., 2016). Briefly, diet-specific Pearson's correlation matrices were calculated for all samples from each diet (*n*=8 per diet) using the psych package for R-software (https://CRAN.R-project.org/package=psych). A high threshold for correlation coefficient |*r*|≥0.8, *P*≤0.05 was chosen based on sample size effects on stability of the main graph theory properties of the network, such as transitivity, density and diameter (Pavlopoulos et al., 2011). The significant correlation matrices were transformed to adjacent lists and visualized in Cytoscape, version 3.5.1 (Shannon et al., 2003). The calculation of network properties was performed using igraph package for R-software (Csardi and Nepusz, 2006).

### Statistical analysis

Sample size calculations were based on our primary outcome, which was the effect of diet treatment on OA histopathology. Based on prior studies, *n*=10 per diet group is estimated to provide >80% power to detect a 30% difference in mean modified Mankin scores with a significance level of *P*=0.05. Secondary outcomes included diet effects on body habitus, serum metabolic biomarkers, and cartilage gene and protein expression. Sample sizes of *n*<10 for serum metabolic biomarkers were due to insufficient sample availability. Cartilage gene and protein samples were collected from ten animals per group. However, samples from two LFLS animals were excluded owing to the presence of tumor-like liver nodules; additional samples from LFHS and HFLS groups were excluded owing to poor sample quality for a final sample size of *n*=8 per group. Histology and body composition data for LFHS and HFLS groups (*n*=10 each) were analyzed from animals included in a prior study (Barboza et al., 2017). An additional 30 animals (*n*=10 per group) were purchased for all additional outcomes. Diet treatment effects were evaluated by one-way ANOVA. Data that did not meet test assumptions for homoscedasticity, even after transformation, were analyzed by Kruskal–Wallis test. Tests showing a significant effect of diet (*P*<0.05) were followed up with multiple-comparison post hoc tests to identify individual group differences as specified in figure legends. Statistical tests were conducted using the software Prism 7.0b for Mac OS X. Data are expressed as mean±s.e.m. unless otherwise stated. *n* indicates animal numbers per group, and sample sizes are provided in figure legends. *P*<0.05 was considered significant. The heatmap in Fig. 5 was created using the ggplot2 package for R.

## Supplementary Material

Supplementary information
