# Supplementary material for: Independent effects of dietary fat and sucrose content on chondrocyte metabolism and osteoarthritis pathology in mice
Source: Dis Model Mech. 2018 Aug 31;11(9):dmm034827. doi: 10.1242/dmm.034827 (PMC6176996; doi:10.1242/dmm.034827)
Supplement: Supplementary information [file dmm-11-034827-s1.pdf]

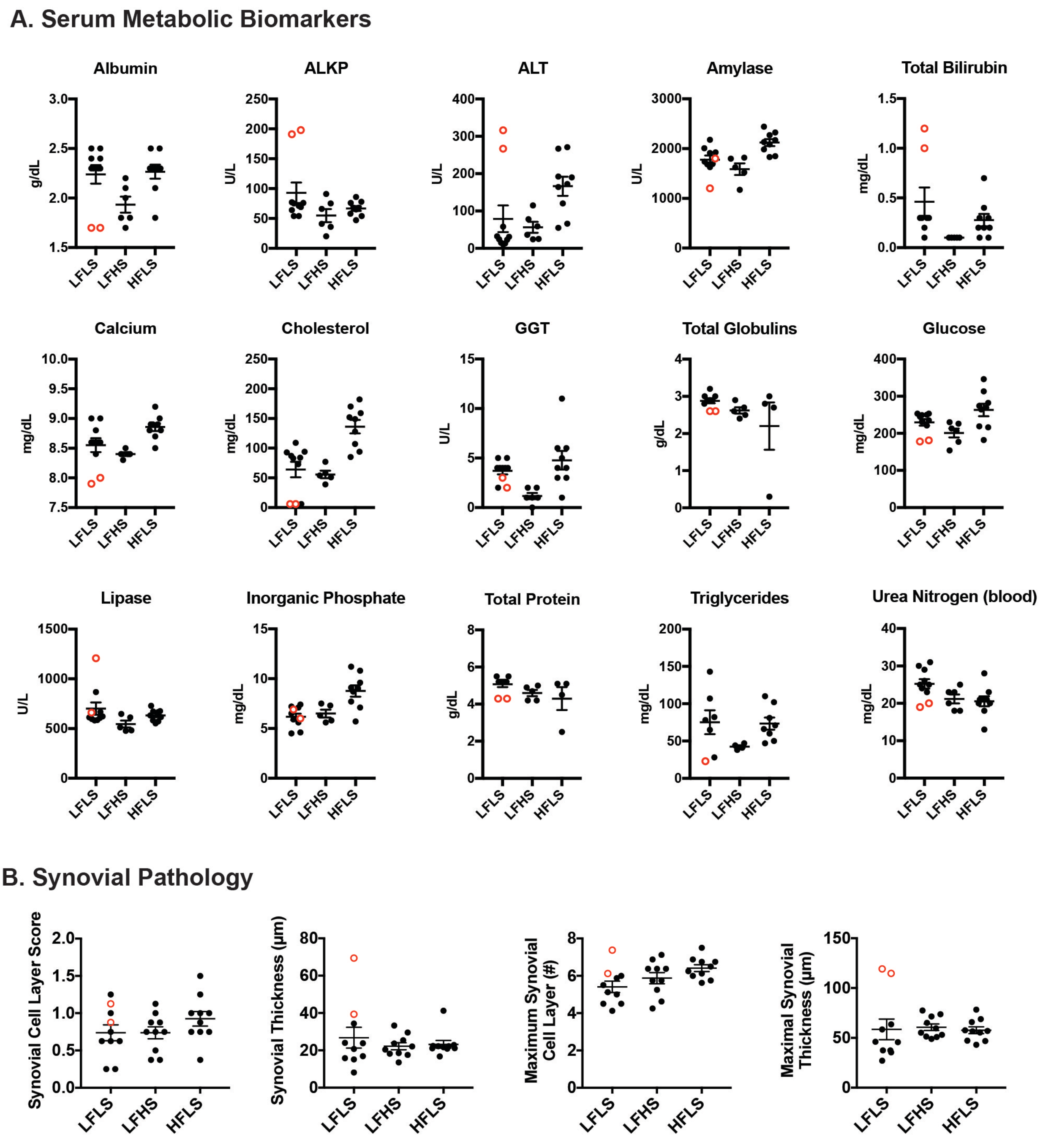

Figure S1: Metabolic and synovial results highlighting excluded outlier data. We observed tumor-like liver nodules during tissue harvest in two LFLS animals (red open circles). A. Metabolic biomarker data from affected animals were identified as outliers based on a ROUT outlier analysis (Q=5%) for Albumin, ALKP, ALT, Total Bilirubin, Glucose, and Lipase (one sample). These results are consistent with impaired liver function; therefore, the data for these animals were excluded from all the metabolic panels. B. Affected animals also showed increased synovial thickness and cellularity, although cartilage and osteophyte results were not altered (data not shown). Thus, only synovial data were excluded from these animals. The time course of liver disease onset in these animals is not known.

Disease Models & Mechanisms • Supplementary information

A. Regions of evaluation and terminology definitions

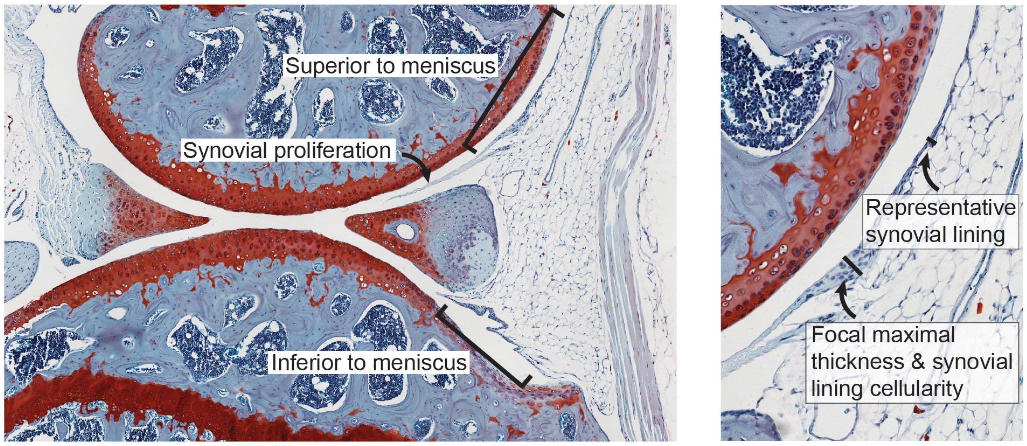

B. Evaluation categories

1) Synovial lining cell layer enlargement (representative region); semi-quantitative scoring (0-3) (see Table S2)
2) Synovial lining thickness (representative region) (µm)
3) Synovial proliferation into cartilage articulating surfaces (anterior region) (0=absent, 1=present)
4) Maximal focal synovial lining cell number (per evaluation region) (# of cells)
5) Maximal focal synovial lining thickness (per evaluation region) (µm)

C. Summary of diet treatment results

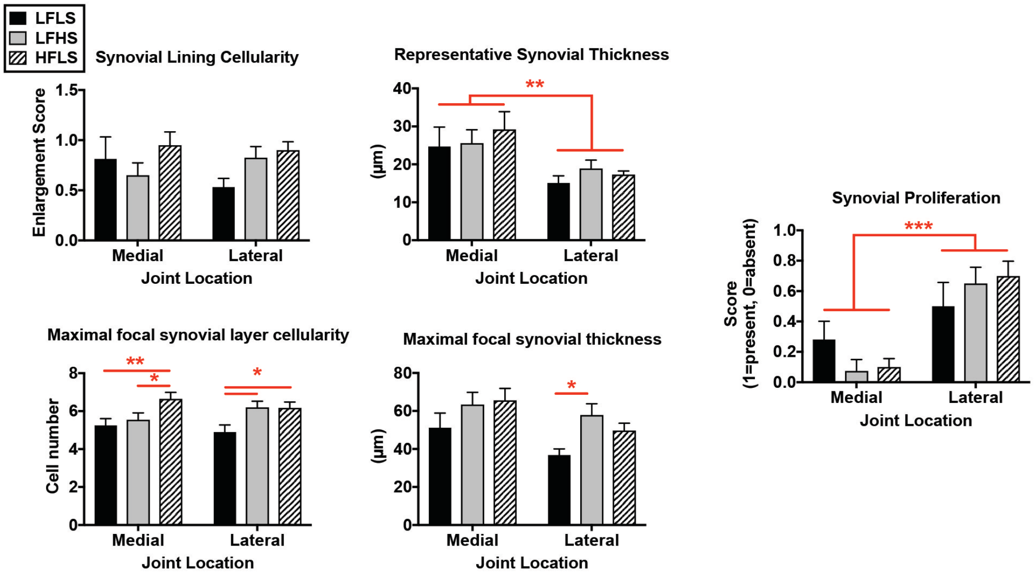

Figure S2: Synovial histopathology assessment and location-specific results. A. The synovium was evaluated for lining layer cellularity and thickening in the joint region superior and inferior to the anterior horn of the meniscus. Due to occasional thickened synovium adjacent to the meniscus, focal maximal values were recorded separately from more distal uniform regions (i.e., representative regions). B. All data were collected in a blinded fashion from medial and lateral compartments (2 sections each). Replicate data averaged to generate one value per animal. C. Bars are mean±s.e.m. Lines over bars indicate significant differences (*P<0.05, **P<0.01, ***P<0.001) by Kruskal-Wallis or repeated-measures two-way ANOVA with FDR-corrected post-hoc tests (0.05). *n*=8 LFLS, *n*=10 LFHS, *n*=10 HFLS.

Disease Models & Mechanisms • Supplementary information

Table S1. Composition of Diets

	LFLS		LFHS		HFLS	
Research Diets, Inc.	#D12450J		#D12450B		#D12492	
	gram%	kcal%	gram%	kcal%	gram%	kcal%
Protein	19.2	20	19.2	20	26	20
Carbohydrate	67.3	70	67.3	70	26	20
Fat	4.3	10	4.3	10	35	60
Total		100		100		
kcal/gm	3.85		3.85		5.24	
Ingredient	gram	kcal	gram	kcal	gram	kcal
Casein, 30 Mesh	200	800	200	800	200	800
L-Cystine	3	12	3	12	3	12
Corn Starch	506	2025	315	1260	0	0
Maltodextrin 10	125	500	35	140	125	500
Sucrose	68.8	275	350	1400	68.8	275
Cellulose, BW200	50	0	50	0	50	0
Soybean Oil	25	225	25	225	25	225
Lard	20	180	20	180	245	2205
Mineral Mix S10026	10	0	10	0	10	0
DiCalcium Phosphate	13	0	13	0	13	0
Calcium Carbonate	5.5	0	5.5	0	5.5	0
Potassium Citrate, 1 H2O	16.5	0	16.5	0	16.5	0
Vitamin Mix V10001	10	40	10	40	10	40
Choline Bitartrate	2	0	2	0	2	0
FD&C Yellow Dye #5	0.04	0	0.05	0	0	0
FD&C Red Dye #40	0	0	0	0	0	0
FD&C Blue Dye #1	0.01	0	0	0	0.05	0
Total	1055.05	4057	1055.05	4057	773.85	4057

Disease Models & Mechanisms • Supplementary information

Table S2. OA Histophatology Scoring Criteria

OARSI cartilage OA pathology for mouse knee joints (Glasson et al., 2010)		
	Grade	Description
	0	Normal
	0.5	Loss of Safranin-O without structural changes
	1	Small fibrillations without loss of cartilage
	2	Vertical clefts down to the layer immediately below the superficial layer and some loss of surface lamina
	3	Verical clefts/erosion to the calcified cartilage extending to <25% of the articular surface
	4	Vertical clefts/erosion to the calcified cartilage extending to 25% - 50% of the articular surface
	5	Vertical clefts/erosion to the calcified cartilage extending to 50% - 75% of the articular surface
	6	Vertical clefts/erosion to the calcified cartilage extending >75% of the articular surface

Modified Mankin cartilage OA pathology for mouse knee joints (Furman et al. 2007)		
Parameter	Grade	Description
**Articular Cartilage Structure**	0	Normal
	1	Undulating articular surface, but no fibrilation
	2	Mild superficial fibrilation involving < half of plateau/condyle
	3	Mild superficial fibrilation involving ≥ half of plateau/condyle
	4	Mild fibrillation/clefts/loss involving up to 1/3 depth of noncalcified articular cartilage thickness in < half of plateau/condyle
	5	Mild fibrillation/clefts/loss involving up to 1/3 depth of noncalcified articular cartilage thickness in ≥ half of plateau/condyle
	6	Moderate fibrillation/clefts/loss involving up to 2/3 depth of noncalcified articular cartilage thickness in < half of plateau/condyle
	7	Moderate fibrillation/clefts/loss involving up to 2/3 depth of noncalcified articular cartilage thickness in ≥ half of plateau/condyle
	8	Severe fibrillation/clefts/loss involving > 2/3 depth of noncalcified articular cartilage thickness in < half of plateau/condyle
	9	Severe fibrillation/clefts/loss involving > 2/3 depth of noncalcified articular cartilage thickness in ≥ half of plateau/condyle
	10	Clefts/loss of articular cartilage through tidemark
	11	Clefts/loss of articular cartilage through to subchondral bone
**Tidemark duplication**	0	none (only one tidemark)
	1	2 tidemarks
	2	>2 tidemarks
	3	no visable tidemark remaining
**Safranin-O staining*** *loss of cartilage treated as severe loss of staining	0	Normal (no loss of staining in non-calcified cartilage)
	1	Moderate loss of staining in up to 1/2 depth of noncalcified cartilage thickness and involving < half of plateau/condyle
	2	Moderate loss of staining up to 1/2 depth of noncalcified cartilage thickness and involving ≥ half of plateau/condyle
	3	Moderate loss of staining in > 1/2 depth of noncalcified cartilage thickness and involving < half of plateau/condyle
	4	Moderate loss of staining in > 1/2 depth of noncalcified cartilage thickness and involving ≥ half of plateau/condyle
	5	Severe loss of staining up to 1/2 depth of noncalcified cartilage thickness and involving < half of plateau/condyle
	6	Severe loss of staining up to 1/2 depth of noncalcified cartilage thickness and involving ≥ half of plateau/condyle
	7	Severe loss of staining in > 1/2 depth of noncalcified cartilage thickness and involving < half of plateau/condyle
	8	Severe loss of staining in > 1/2 depth of non-calcified cartilage thickness and involving ≥ half of plateau/condyle
**Calficied cartilage**	0	0-5 hypertrophic chondrocytes
	1	6-10 hypertrophic chondrocytes
	2	>10 hypertrophic chondrocytes

Osteophyte severity (Kraus et al. 2010)		
	Grade	Description
	0	Normal
	1	Slight protrusion resulting in a right-angle between articular surface and the lateral/medial tibial condyle
	2	Clear protrusion of the lateral/medial tibial condyle
	3	Severe protrusion of lateral/medial condyle resulting in an inferior-oriented capsular attachment surface (e.g., "bucket handle")

Synovial lining cell layer enlargement (modified from Lewis et al. 2011)		
	Grade	Description
	0	Thickness 1-2 cells
	1	Thickness 2-4 cells
	2	Thickness 4-6 cells
	3	Thickness ≥7 cells

Glasson, S. S., Chambers, M. G., van den Berg, W. B., & Little, C. B. (2010). The OARSI histopathology initiative - recommendations for histological assessments of osteoarthritis in the mouse. Osteoarthritis and Cartilage, 18(S3), S17-S23.

Furman, B. D., Strand, J., Hembree, W. C., Ward, B. D., Guilak, F., & Olson, S. A. (2007). Joint degeneration following closed intraarticular fracture in the mouse knee: A model of posttraumatic arthritis. Journal of Orthopaedic Research, 25(5), 578–592.

Kraus, V. B., Huebner, J. L., DeGroot, J., & Bendele, A. (2010). The OARSI histopathology initiative - recommendations for histological assessments of osteoarthritis in the guinea pig. Osteoarthritis Cartilage, 18(S3), S35–S52.

Lewis, J. S., Hembree, W. C., Furman, B. D., Tippets, L., Cattel, D., Huebner, J. L., et al. (2011). Acute joint pathology and synovial inflammation is associated with increased intra-articular fracture severity in the mouse knee. Osteoarthritis and Cartilage / OARS, Osteoarthritis Research Society, 19(7), 864–873.

Disease Models & Mechanisms • Supplementary information

Table S3. Diet-specific effects on cartilage gene expression

Gene name	NCBI Ref. Seq.	LFLS	LFHS	HFLS	*P*-value
Acc	NM_133360	0.013±0.0095	0.0079±0.0012	0.0074±0.00068	NS
Adamts5	NM_011782	0.0095±0.0040	0.0088±0.0021	0.010±0.0044	NS
Acan	NM_007424	0.070±0.046	0.066±0.021	0.082±0.034	NS
Prkaa1	NM_001013367	0.015±0.0032	0.012±0.0018	0.013±0.0017	NS
Bcl2	NM_009741	0.0073±0.0032	0.0049±0.0014	0.0064±0.0023	NS
Casp1	NM_009807	0.019±0.0059	0.016±0.0019	0.019±0.0035	NS
Ddit3	NM_007837	0.024±0.0040a	0.014±0.0033b	0.015±0.0051b	p<0.0001
Clpp	NM_017393	0.00032±0.00015a	0.00014±4.6e-005b	0.00016±4.5e-005b	p=0.0012
Clpx	NM_011802	0.024±0.0058a	0.017±0.0036b	0.020±0.0031a,b	p=0.0320
Col10a1	NM_009925	0.21±0.17	0.13±0.057	0.17±0.089	NS
Col2a1	NM_031163	0.18±0.11	0.22±0.18	0.18±0.079	NS
Ptgs2	NM_011198	0.00086±0.00050	0.0011±0.00065	0.00080±0.00039	NS
Cpt1a	NM_001876	0.013±0.0021a	0.013±0.0049a	0.019±0.0026b	p=0.0023
Esrra	NM_007953	0.0033±0.0017	0.0030±0.00068	0.0037±0.0014	NS
Foxo3	NM_019740	0.086±0.011a	0.049±0.015b	0.062±0.017b	p=0.0002
Hif1a	NM_010431	0.029±0.011	0.029±0.0066	0.031±0.0063	NS
Epas1	NM_010137	0.032±0.011	0.023±0.0035	0.029±0.011	NS
Hsp10	NM_008303	0.058±0.011a	0.041±0.0080b	0.039±0.0094b	p=0.0014
Hspd1	NM_010477	0.039±0.0037	0.034±0.0069	0.032±0.0083	NS
Hspa1b	NM_010478	0.0089±0.0054	0.0087±0.0057	0.0053±0.0035	NS
Hsp90	NM_010480	0.33±0.035a	0.27±0.054a,b	0.25±0.073b	p=0.0308
Idh2	NM_173011	0.035±0.0095	0.028±0.0044	0.032±0.0057	NS
Map1lc3a	NM_025735	0.013±0.0060	0.012±0.0046	0.013±0.0033	NS
Acadl	NM_007381	0.069±0.018	0.058±0.0085	0.059±0.011	NS
Lonp1	NM_028782	0.013±0.0032a	0.011±0.0013b	0.012±0.0017a,b	p=0.0148
Mmp13	NM_008607	0.11±0.041	0.11±0.071	0.11±0.064	NS
Mmp9	NM_013599	0.39±0.10	0.43±0.11	0.41±0.097	NS
Pmpca	NM_173180	0.010±0.0032	0.0094±0.0012	0.0099±0.0010	NS
Mtif2	NM_133767	0.011±0.0019a	0.0083±0.0015b	0.0094±0.0016a,b	p=0.0197
Nampt	NM_021524	0.037±0.0092	0.032±0.0027	0.036±0.0068	NS
Nfkb1	NM_008689	0.038±0.0091	0.031±0.0067	0.034±0.0054	NS
Nos2	NM_010927	0.00013±6.0e-005	0.00021±0.00023	0.00013±0.00012	NS
Nox4	NM_015760	0.0027±0.0027	0.0021±0.0013	0.0018±0.0018	NS
Pdk4	NM_013743	0.0094±0.0089	0.0078±0.0061	0.0074±0.0033	NS
Pfkm	NM_021514	0.051±0.042	0.044±0.040	0.022±0.012	NS
Ppargc1a	NM_008904	0.00044±0.00035	0.00030±0.00021	0.00020±0.00011	NS
Rela	NM_009045	0.0084±0.0021	0.0074±0.0017	0.0082±0.0014	NS
Sirt1	NM_019812	0.022±0.0051	0.016±0.0042	0.020±0.0044	NS
Sirt3	NM_022433	0.0079±0.0021	0.0066±0.0016	0.0070±0.0014	NS
Sod1	NM_011434	0.12±0.023a	0.072±0.020b	0.085±0.031b	p=0.0010
Sod2	NM_013671	0.051±0.024a	0.030±0.011b	0.029±0.011b	p=0.0186
Xbp1	NM_013842	0.075±0.029	0.063±0.018	0.059±0.026	NS

Gene expression (2^(-dCt)) of cartilage harvested from mice fed a LFLS (low fat/low sucrose), LFHS (low fat/high sucrose), or HFLS (high fat/low sucrose) diet was measured using quantitative PCR. Results were compared among the three diet groups (*n*=8 per diet). Data were log-transformed for one-way ANOVA testing or analyzed by Kruskal-Wallace to meet statistical model assumptions. Genes that showed significant diet differences ($P< 0.05$) were further analyzed by Tukey's post-hoc test (ANOVA) or Dunn's multiple comparisons test (K-W). Diets not sharing the letters "a" and "b" are significantly different (P-adj<0.05). Values are mean±s.d.

Table S4. Diet-specific effects on cartilage protein abundance

Name	Abbreviation	LFLS	LFHS	HFLS	*P*-value
ATP-binding cassette, sub-family D (ALD), member 3	ABCD3	1.43±0.53b	0.87±0.26a	1.15±0.33a,b	0.0458
acetyl-Coenzyme A acyltransferase 1A_B	ACAA1A_B	1.26±0.3	0.85±0.28	1.21±0.37	NS
acetyl-Coenzyme A acyltransferase 2 (mitochondrial 3-oxoacyl-Coenzyme A thiolase)	ACAA2	0.84±0.15b	0.9±0.14b	1.12±0.17a	0.0069
acyl-Coenzyme A dehydrogenase, long-chain	ACADL	1.19±0.26	0.92±0.2	1.17±0.28	NS
acyl-Coenzyme A dehydrogenase, medium chain	ACADM	1.49±0.53	0.98±0.32	1.21±0.18	NS
acyl-Coenzyme A dehydrogenase, short chain	ACADS	0.78±0.37	0.82±0.31	1.05±0.41	NS
acyl-Coenzyme A dehydrogenase, very long chain	ACADVL	0.87±0.11	0.9±0.18	1.03±0.12	NS
aconitase 2, mitochondrial	ACO2	1.45±0.31b	1.06±0.36a	1.03±0.2a	0.0241
acyl-CoA synthetase long-chain family member 1	ACSL1	1.07±0.36	0.94±0.23	1.03±0.23	NS
aldo-keto reductase family 1, member B3 (aldose reductase)	AKR1B1	0.7±0.29b	1.12±0.24a	1±0.23a,b	0.0179
albumin_murine	ALB_M	1.82±0.5b	0.93±0.4a	1.32±0.5a,b	0.0073
aldehyde dehydrogenase 2, mitochondrial	ALDH2	1.46±0.43b	0.92±0.27a	1.17±0.2a,b	0.0164
aldolase A, fructose-bisphosphate	ALDOA	1.24±0.44	1.24±0.4	0.87±0.18	NS
ATPase, Ca++ transporting, cardiac muscle, fast twitch 1	ATP2A1	1.44±1.36	1.84±1.35	0.87±0.58	NS
ATPase, Ca++ transporting, cardiac muscle, slow twitch 2	ATP2A2	1.09±0.46	1.44±0.81	0.87±0.31	NS
ATP synthase, H+ transporting, mitochondrial F1 complex, alpha subunit 1	ATP5A1	0.69±0.19b	1.26±0.41a	0.98±0.09a,b	0.0029
ATP synthase, H+ transporting mitochondrial F1 complex, beta subunit	ATP5B	0.64±0.15b	1.18±0.43a	0.98±0.16a,b	0.0057
3-hydroxybutyrate dehydrogenase, type 1	BDH1	1.41±0.78	1.04±0.35	0.9±0.17	NS
calreticulin	CALR	1.05±0.38	0.85±0.18	1.09±0.12	NS
catalase	CAT	1.24±0.41b	0.78±0.3a	1.2±0.27a,b	0.03
CD36 antigen	CD36	1.8±1.02	0.94±0.4	1.55±1.24	NS
creatine kinase, muscle	CKM	1.16±0.47	1.33±0.55	0.82±0.3	NS
creatine kinase, mitochondrial 2	CKMT2	1.23±0.76	1.22±0.49	0.99±0.3	NS
carnitine palmitoyltransferase 1b, muscle	CPT1B	1.28±0.67	1.23±0.57	0.94±0.3	NS
carnitine palmitoyltransferase 2	CPT2	0.92±0.36	0.84±0.24	1.18±0.22	NS
carnitine acetyltransferase	CRAT	1.13±0.51	0.99±0.29	0.95±0.29	NS
crystallin, alpha B	CRYAB	1.98±1.26	1.55±0.81	1.14±0.61	NS
citrate synthase	CS	1.07±0.27	1.02±0.18	1.04±0.1	NS
2,4-dienoyl CoA reductase 1, mitochondrial	DECR1	0.89±0.16	1.11±0.26	1.11±0.27	NS
dihydrolipoamide S-acetyltransferase (E2 component of pyruvate dehydrogenase complex)	DLAT	0.8±0.15	1.09±0.35	0.97±0.19	NS
dihydrolipoamide dehydrogenase	DLD	1.28±0.2b	1.1±0.22a,b	1±0.14a	0.0341
dihydrolipoamide S-succinyltransferase (E2 component of 2-oxo-glutarate complex)	DLST	0.79±0.18	0.97±0.25	1.02±0.13	NS
enoyl coenzyme A hydratase 1, peroxisomal	ECH1	1.02±0.28a,b	0.8±0.17b	1.28±0.24a	0.0041

Disease Models & Mechanisms • Supplementary information

enoyl Coenzyme A hydratase, short chain, 1, mitochondrial	ECHS1	0.99±0.16	0.98±0.11	1±0.14	NS
enoyl-Coenzyme A delta isomerase 1	ECI1	0.69±0.18b	1.17±0.27a	0.96±0.2a,b	0.0021
enoyl-Coenzyme A delta isomerase 2	ECI2	0.71±0.16b	0.86±0.22a,b	1.12±0.3a	0.012
enolase 1, alpha non-neuron	ENO1	1.07±0.25a,b	0.86±0.18b	1.14±0.16a	0.0478
enolase 3, beta muscle	ENO3	1.65±0.81	1.54±0.69	0.82±0.4	NS
electron transferring flavoprotein, alpha polypeptide	ETFA	1.05±0.21	1.04±0.14	1.07±0.25	NS
electron transferring flavoprotein, beta polypeptide	ETFB	0.98±0.55	1.3±0.82	1.05±0.33	NS
electron transferring flavoprotein, dehydrogenase	ETFDH	1.02±0.3	0.95±0.26	1.01±0.15	NS
fatty acid binding protein 4, adipocyte	FABP4	1.14±0.58	0.94±0.29	1.11±0.35	NS
fumarate hydratase 1	FH1	1.47±0.36b	1.01±0.24a	1.13±0.34a,b	0.037
glyceraldehyde-3-phosphate dehydrogenase	GAPDH	1.1±0.2	1.09±0.23	0.91±0.12	NS
glutamate dehydrogenase 1	GLUD1	1.41±0.41	0.99±0.31	1.25±0.55	NS
glutamic-oxaloacetic transaminase 1, soluble	GOT1	1.28±0.43	1.24±0.39	0.91±0.12	NS
glutamatic-oxaloacetic transaminase 2, mitochondrial	GOT2	1.16±0.32	0.98±0.26	1±0.13	NS
glutathione peroxidase 1	GPX1	1.39±0.28a	0.82±0.26b	1.29±0.29a	0.002
glutathione peroxidase 4	GPX4	1.5±0.43	1.03±0.41	1.07±0.3	NS
glutathione reductase	GSR	1.14±0.43	0.9±0.18	1.13±0.25	NS
glutathione S-transferase, mu 1	GSTM1	0.94±0.11	1.03±0.27	1.06±0.23	NS
glutathione S-transferase, pi 1	GSTP1	0.98±0.34	1.06±0.3	1.05±0.37	NS
hydroxyacyl-Coenzyme A dehydrogenase	HADH	0.94±0.27	1.03±0.2	1.15±0.25	NS
hydroxyacyl-Coenzyme A dehydrogenase/3-ketoacyl-Coenzyme A thiolase/enoyl-Coenzyme A hydratase (trifunctional protein), alpha subunit	HADHA	1.35±0.33a	0.87±0.27b	1.24±0.18a	0.008
hydroxyacyl-Coenzyme A dehydrogenase/3-ketoacyl-Coenzyme A thiolase/enoyl-Coenzyme A hydratase (trifunctional protein), beta subunit	HADHB	0.83±0.17	0.93±0.13	1.05±0.17	NS
hexokinase 1	HK1	1.35±0.37	0.93±0.28	1.15±0.31	NS
hydroxysteroid (17-beta) dehydrogenase 4	HSD17B4	0.97±0.28	0.87±0.15	1.1±0.19	NS
heat shock protein 90, beta (Grp94), member 1	HSP90B1	1.4±0.62	0.88±0.21	1.24±0.41	NS
heat shock protein 1A	HSPA1A	1.27±0.47	0.88±0.21	1.13±0.1	NS
heat shock protein 5	HSPA5	1.16±0.33	0.85±0.26	1.09±0.38	NS
heat shock protein 9	HSPA9	1.32±0.49	0.89±0.25	1.19±0.19	NS
isocitrate dehydrogenase 1 (NADP+), soluble	IDH1	1.09±0.28	0.89±0.28	1.22±0.2	NS
isocitrate dehydrogenase 2 (NADP+), mitochondrial	IDH2	1.19±0.26	0.93±0.3	1.16±0.17	NS
isocitrate dehydrogenase 3 (NAD+) alpha	IDH3A	1.32±0.52	1.13±0.39	1.04±0.2	NS
isocitrate dehydrogenase 3 (NAD+) beta	IDH3B	0.78±0.38b	1.33±0.44a	0.92±0.18a,b	0.0202
isocitrate dehydrogenase 3 (NAD+), gamma	IDH3G	1.18±0.43	1.05±0.24	1±0.14	NS
lactate dehydrogenase A	LDHA	0.8±0.27	1.13±0.3	0.99±0.28	NS
lactate dehydrogenase B	LDHB	1.35±0.26b	0.98±0.16a	1.01±0.18a	0.0048
lon peptidase 1, mitochondrial	LONP1	1.42±0.36	1.02±0.34	1.21±0.37	NS

Disease Models & Mechanisms • Supplementary information

malate dehydrogenase 2, NAD (mitochondrial)	MDH2	0.92±0.24	1.01±0.21	0.93±0.15	NS
methionine sulfoxide reductase A	MSRA	1.01±0.28	0.88±0.25	1.16±0.3	NS
NADH dehydrogenase (ubiquinone) Fe-S protein 1	NDUFS1	1.2±0.35	0.94±0.33	1.03±0.13	NS
NADH dehydrogenase (ubiquinone) flavoprotein 1	NDUFV1	1.09±0.24	1±0.37	0.94±0.25	NS
oxoglutarate (alpha-ketoglutarate) dehydrogenase (lipoamide)	OGDH	1.28±0.34	0.97±0.27	1.09±0.19	NS
pyruvate carboxylase	PC	2.68±1.71b	1.25±0.55a	0.92±0.3a	0.0114
pyruvate dehydrogenase E1 alpha 1	PDHA1	1.33±0.41	1.1±0.34	1.02±0.17	NS
phosphofructokinase, liver, B-type	PFKL	1.28±0.29a	0.88±0.14b	1.24±0.32a	0.0191
phosphofructokinase, muscle	PFKM	1.36±0.76	1.6±0.9	0.87±0.39	NS
phosphoglycerate mutase 2	PGAM2	1.52±0.78	1.39±0.71	0.75±0.26	NS
phosphoglycerate kinase 1	PGK1	0.96±0.11	1.12±0.25	0.97±0.09	NS
pyruvate kinase, muscle 2	PKM2	1.17±0.08	1.06±0.3	0.97±0.14	NS
peroxiredoxin 1	PRDX1	1.53±0.4b	0.91±0.24a	1.24±0.29a,b	0.0052
peroxiredoxin 2	PRDX2	2.02±0.85b	0.89±0.23a	1.46±0.5a,b	0.0063
peroxiredoxin 3	PRDX3	1.02±0.26	1.03±0.31	1.03±0.18	NS
peroxiredoxin 5	PRDX5	0.83±0.19	0.98±0.36	1.26±0.69	NS
peroxiredoxin 6	PRDX6	1.11±0.14	0.92±0.13	1.18±0.27	NS
protein kinase, cAMP dependent, catalytic, alpha	PRKACA	1.24±0.29	0.96±0.22	1.23±0.24	NS
brain glycogen phosphorylase	PYGB	1.69±0.55b	0.98±0.4a	1.17±0.24a,b	0.0123
muscle glycogen phosphorylase	PYGM	1.49±1.11	1.82±1.37	1.03±0.78	NS
succinate dehydrogenase complex, subunit A, flavoprotein (Fp)	SDHA	1.3±0.38	1.01±0.24	1.06±0.19	NS
succinate dehydrogenase complex, subunit B, iron sulfur (Ip)	SDHB	1.21±0.37	1.01±0.32	1.12±0.26	NS
solute carrier family 25 (mitochondrial carrier, citrate carrier), member 1	SLC25A1	0.84±0.56	1.63±1.41	0.86±0.29	NS
superoxide dismutase 1, soluble	SOD1	1.35±0.37b	0.86±0.21a	1.09±0.31a,b	0.0253
superoxide dismutase 2, mitochondrial	SOD2	1.5±0.26b	1±0.32a	1.02±0.2a	0.0025
succinate-Coenzyme A ligase, ADP-forming, beta subunit	SUCLA2	0.76±0.35	1.07±0.29	0.93±0.17	NS
transaldolase 1	TALDO1	1.13±0.39	0.85±0.32	1.11±0.37	NS
transketolase	TKT	1.14±0.45	0.89±0.2	1.11±0.19	NS
triosephosphate isomerase 1	TPI	1.13±0.29	1.17±0.37	0.92±0.17	NS
thioredoxin 1	TXN1	1.1±0.23	0.93±0.26	1.05±0.27	NS
thioredoxin reductase 1	TXNRD1	1.22±0.35b	0.86±0.19a	1.13±0.17a,b	0.0356
ubiquinol-cytochrome c reductase core protein 1	UQCRC1	0.98±0.15	1.02±0.21	1.02±0.16	NS

Protein data obtained by selected reaction monitoring (SRM) mass spectrometry. Values were normalized to a BSA internal standard and cellular reference protein MDH1. Samples were analyzed in two separate runs (Run 1: LFHS (n=3) and HFLS (n=5); Run 2: LFLS (n=8), LFHS (n=5), HFLS (n=3)), and data were normalized to the median response of LFHS and HFLS samples for each run. The integrated data were used for statistical comparison among the three diet groups (n=8 per diet) by one-way ANOVA. The enzymes that showed significant differences (P<0.05) were analyzed by Tukey post-hoc test. Diets not sharing the letters "a" and "b" are significantly different (P-adj<0.05). Values are mean±s.d.

Table S5. Control and high fat diet compositions in obese rodent models of idiopathic OA

Species (Sex)	Control Diet Composition	High Fat Diet Composition	Matched Diets?	Age begin diet & diet duration	OA phenotype(s)	Ref.
Rat (M)	Lab Diet 5001<br>CHO (57.9 %kcal)<br>Sucrose (3.2%), Starch (22%)<br>Fat (13.4 %kcal)<br>Lard, fish meal	#102412, Dyets Inc.<br>CHO (45 %kcal)<br>Sucrose (100%)<br>Fat (40 %kcal)<br>Lard, soybean oil	No	8-12 weeks, 28 weeks	Cartilage pathology, synovial fluid inflammation	1
Rat (M)	NIH-31<br>CHO (~74 %kcal)<br>N/A<br>Fat (~9 %kcal)<br>Fish meal, soybean oil	NIH-31 (adult-onset obesity)<br>CHO (~74 %kcal)<br>N/A<br>Fat (~9 %kcal)<br>Fish meal, soybean oil	Yes	Weaning, 30 months	Cartilage and meniscus pathology, synovial thickening	2
Rat (M)	Custom<br>CHO (N/A)<br>Starch (57%)<br>Fat (N/A)	Custom<br>CHO (N/A)<br>Fructose, sweetened condensed milk<br>Fat (N/A)<br>Beef tallow (20%)	No	9-10 weeks, 16 weeks	Cartilage pathology, reduced sub-chondral bone fraction, synovial inflammation	3,4
Mouse (F)	#D12450B, Research Diets Inc<br>CHO (70 %kcal)<br>Sucrose (33%), Starch (30%)<br>Fat (10 %kcal)<br>Lard (2%), soybean oil (2.4%)	#D12451, Research Diets Inc<br>CHO (35 %kcal)<br>Sucrose (20%), Starch (8%)<br>Fat (45 %kcal)<br>Lard (21%), soybean oil (3%)	Yes	9 weeks, 45 weeks	Cartilage pathology	5
Mouse (M)	#CE-2, CLEA Japan Inc<br>CHO (58 %kcal)<br>N/A<br>Fat (12 %kcal)<br>Cereal germ, soybean oil	#HFD32, CLEA Japan Inc<br>CHO (23 %kcal)<br>Sucrose (7%)<br>Fat (57 %kcal)<br>Beef tallow (16%), safflower oil (20%)	No	7 weeks, 12 weeks	Cartilage pathology, osteophytes, synovial thickening	6
Mouse (M)	#D12450B, Research Diets Inc<br>CHO (70 %kcal)<br>Sucrose (33%), Starch (30%)<br>Fat (10 %kcal)<br>Lard (2%), soybean oil (2.4%)	#D12492, Research Diets Inc<br>CHO (20 %kcal)<br>Sucrose (9%), Starch (0%)<br>Fat (60 %kcal)<br>Lard (32%), soybean oil (3%)	Yes	5 weeks, 24 weeks	Osteophytes, synovial inflammation	7
Mouse (M)	#D12450B, Research Diets Inc<br>CHO (70 %kcal)<br>Sucrose (33%), Starch (30%)<br>Fat (10 %kcal)<br>Lard (2%), soybean oil (2.4%)	#D12492, Research Diets Inc<br>CHO (20 %kcal)<br>Sucrose (9%), Starch (0%)<br>Fat (60 %kcal)<br>Lard (32%), soybean oil (3%)	Yes	6 weeks, 20-46 weeks	Osteophytes (20 weeks), cartilage pathology (46 weeks)	8
Mouse (M)	Rodent Chow 5001, Purina Inc<br>CHO (~60 %kcal)<br>Sucrose (3.7%), Starch (32%)<br>Fat (13.5 %kcal)<br>Lard, fish meal	#D12492, Research Diets Inc<br>CHO (20 %kcal)<br>Sucrose (9%), Starch (0%)<br>Fat (60 %kcal)<br>Lard (32%), soybean oil (3%)	No	12 weeks, 12 weeks	Cartilage pathology, increased sub-chondral bone thickness	9
Mouse (M)	#V1534 Chow, Ssniff GmbH<br>CHO (67 %kcal)<br>Sucrose (5.4%), Starch (36%)<br>Fat (9 %kcal)<br>N/A	#D12451, Ssniff Speziäldiaten<br>CHO (35 %kcal)<br>Sucrose (20%), Starch (8%)<br>Fat (45 %kcal)<br>Lard (21%), soybean oil (3%)	No	12 weeks, 24 weeks	Osteophytes (variable), synovial inflammation (variable)	10
Mouse, Human CRP Tg (M/F)	#V1534 Chow, Ssniff GmbH<br>CHO (67 %kcal)<br>Sucrose (5.4%), Starch (36%)<br>Fat (9 %kcal)<br>N/A	#D12451, Ssniff Speziäldiaten<br>CHO (35 %kcal)<br>Sucrose (20%), Starch (8%)<br>Fat (45 %kcal)<br>Lard (21%), soybean oil (3%)	No	12-16 weeks, 38 weeks	Cartilage pathology (male only)	11

M = male; F = female. CHO = carbohydrate. Diet compositions based on online diet data sheets obtained in March 2018; potential changes from time of study completion were not evaluated. Unless specified, % is by weight.

Table S5 References

1. Collins KH, Paul HA, Reimer RA, Seerattan RA, Hart DA, Herzog W. Relationship between inflammation, the gut microbiota, and metabolic osteoarthritis development: studies in a rat model. *Osteoarthritis Cartilage*. 2015;23(11):1989-1998. doi:10.1016/j.joca.2015.03.014.

2. Fu Y, Kinter M, Hudson J, et al. Aging Promotes Sirtuin 3–Dependent Cartilage Superoxide Dismutase 2 Acetylation and Osteoarthritis. *Arthritis Rheumatol*. 2016;68(8):1887-1898. doi:10.1002/art.39618.

3. Sekar S, Shafie SR, Prasadam I, et al. Saturated fatty acids induce development of both metabolic syndrome and osteoarthritis in rats. *Sci Rep*. 2017;7:46457. doi:10.1038/srep46457.

4. Sun AR, Panchal SK, Friis T, et al. Obesity-associated metabolic syndrome spontaneously induces infiltration of pro-inflammatory macrophage in synovium and promotes osteoarthritis. *PLoS ONE*. 2017;12(8):e0183693. doi:10.1371/journal.pone.0183693.

5. Griffin TM, Fermor B, Huebner JL, et al. Diet-induced obesity differentially regulates behavioral, biomechanical, and molecular risk factors for osteoarthritis in mice. *Arthritis Res Ther*. 2010;12(4):R130. doi:10.1186/ar3068.

6. Aibibula Z, Ailixiding M, Iwata M, et al. Xanthine oxidoreductase activation is implicated in the onset of metabolic arthritis. *Biochemical and Biophysical Research Communications*. 2016;472(1):26-32. doi:10.1016/j.bbrc.2016.02.039.

7. Hamada D, Maynard R, Schott E, et al. Suppressive Effects of Insulin on Tumor Necrosis Factor–Dependent Early Osteoarthritic Changes Associated With Obesity and Type 2 Diabetes Mellitus. *Arthritis Rheumatol*. 2016;68(6):1392-1402. doi:10.1002/art.39561.

8. Barboza E, Hudson J, Chang W-P, et al. Profibrotic Infrapatellar Fat Pad Remodeling Without M1 Macrophage Polarization Precedes Knee Osteoarthritis in Mice With Diet-Induced Obesity. *Arthritis Rheumatol*. 2017;69(6):1221-1232. doi:10.1002/art.40056.

9. Griffin TM, Huebner JL, Kraus VB, Yan Z, Guilak F. Induction of osteoarthritis and metabolic inflammation by a very high-fat diet in mice: Effects of short-term exercise. *Arthritis Rheum*. 2012;64(2):443-453. doi:10.1002/art.33332.

10. Kozijn AE, Gierman LM, van der Ham F, et al. Variable cartilage degradation in mice with diet-induced metabolic dysfunction: food for thought. *Osteoarthritis Cartilage*. November 2017:1-13. doi:10.1016/j.joca.2017.10.010.

11. Gierman LM, van der Ham F, Koudijs A, et al. Metabolic stress-induced inflammation plays a major role in the development of osteoarthritis in mice. *Arthritis Rheum*. 2012;64(4):1172-1181. doi:10.1002/art.33443.
